# Two-coordinate group 14 element(ii) hydrides as reagents for the facile, and sometimes reversible, hydrogermylation/hydrostannylation of unactivated alkenes and alkynes†
†Electronic supplementary information (ESI) available: Experimental procedures and characterisation data for all new compounds, full details of the computational studies. Crystal data, details of data collections and refinements. CCDC 1422725–1422742. For ESI and crystallographic data in CIF or other electronic format see DOI: 10.1039/c5sc03376d


**DOI:** 10.1039/c5sc03376d

**Published:** 2015-09-22

**Authors:** Terrance J. Hadlington, Markus Hermann, Gernot Frenking, Cameron Jones

**Affiliations:** a School of Chemistry , Monash University , PO Box 23 , VIC 3800 , Australia . Email: cameron.jones@monash.edu ; http://www.monash.edu/science/research-groups/chemistry/jonesgroup; b Fachbereich Chemie , Philipps-Universität Marburg , 35032 , Marburg , Germany . Email: frenking@chemie.uni-marburg.de

## Abstract

Reactions of the solution stable, two-coordinate hydrido-tetrylenes, :E(H)(L^†^) (E = Ge or Sn; L^†^ = –N(Ar^†^)(SiPr^i^_3_); Ar^†^ = C_6_H_2_{C(H)Ph_2_}_2_Pr^i^-2,6,4), with a variety of unactivated cyclic and acyclic alkenes, and one internal alkyne, lead to the rapid and regiospecific hydrometallation of the unsaturated substrate at ambient temperature. The products of the reactions, [L^†^E(C_2_H_4_R)] (E = Ge or Sn, R = H, Ph or Bu^*t*^), [L^†^E{CH(CH_2_)_3_(CH_2_)_*n*_}] (E = Ge, *n* = 1, 2 or 3; E = Sn, *n* = 1) and [L^†^E{C(Ph)=C(H)(Me)}], include the first structurally characterised examples of two-coordinate amido/alkyl germylenes and stannylenes. The cycloalkene hydrometallation reactions are cleanly reversible under ambient conditions, a process which computational and experimental van't Hoff analyses suggest proceeds *via* β-hydride elimination from the metal coordinated cycloalkyl ligand. Similarly, the reactions of :Ge(H)(L^†^) with 1,5-cyclooctadiene and 2-methyl-2-butene, both likely proceed *via* β-hydride elimination processes, leading to the clean isomerisation of the alkene involved, and its subsequent hydrogermylation, to give [L^†^Ge(2-cyclooctenyl)] and [L^†^Ge{C_2_H_4_C(H)Me_2_}], respectively. Reactions of [L^†^GeEt] and [L^†^Ge(C_5_H_9_)] with the protic reagents, HCl, NH_3_ and EtOH, lead to oxidative addition to the germanium(ii) centre, and formation of the stable chiral germanium(iv) complexes, [L^†^Ge(C_5_H_9_)(H)Cl] and [L^†^Ge(Et)(H)R] (R = NH_2_ or OEt). In contrast, related reactions between [L^†^SnEt] and Bu^*t*^OH or TEMPOH (TEMP = 2,2,6,6-tetramethylpiperidinyl) proceed *via* ethane elimination, affording the tin(ii) products, [L^†^SnR] (R = OBu^*t*^ or OTEMP). In addition, the oxidation of [L^†^Ge(C_6_H_11_)] and [L^†^Sn(C_2_H_4_Bu^*t*^)] with O_2_ yields the oxo-bridged metal(iv) dimers, [{L^†^(C_6_H_11_)Ge(μ-O)}_2_] and [{L^†^(Bu^*t*^C_2_H_4_)Sn(μ-O)}_2_], respectively.

## Abstract 2

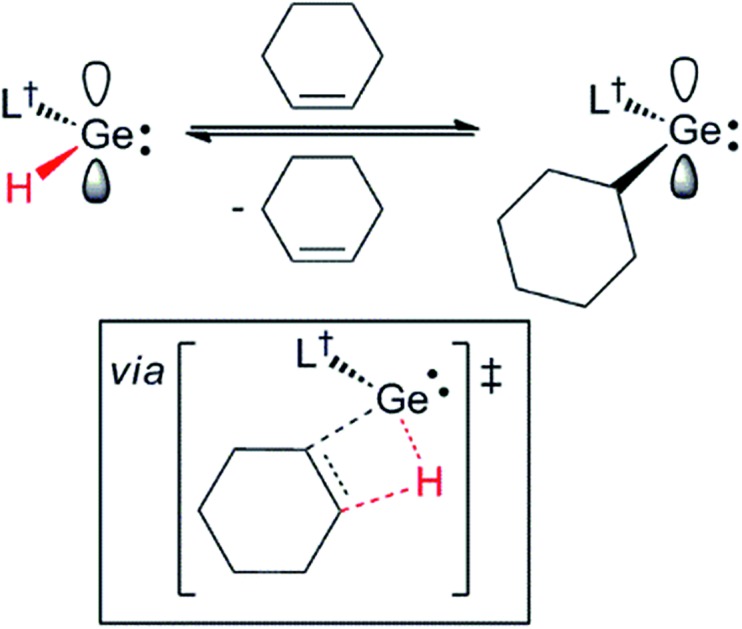
The ambient temperature hydrometallations of a variety of unactivated alkene and alkyne substrates using two-coordinate hydrido-tetrylenes, :E(H)(L^†^) (E = Ge or Sn; L^†^ = extremely bulky amide), are reported.

## Introduction

The 1,2-addition of element-hydrogen bonds across the carbon–carbon unsaturations of alkenes and alkynes is of immense importance to organic synthesis. In this respect, and since Brown's seminal work on the hydroboration of alkenes in the 1950's,^1^ boranes have become the reagent of choice for the reduction of olefins and alkynes.^2^ One of the primary reasons for the efficacy of such hydroborations, is that electron deficient, three-coordinate boranes (R_2_BH) possess an empty p-orbital which is thought to allow the formation of a loose π-complex with the unsaturated substrate, prior to its insertion into the polar ^δ+^B–H^δ–^ linkage.^3^ This mechanism has also been used to explain the typically observed *cis*-/*anti*-Markovnikov addition of boranes to unsaturated hydrocarbons. While much less studied than boranes, a variety of electron deficient, polar hydride complexes of aluminium, the heavier group 13 metals,^2^ and the s-4 and early d-block metals,^5^ have additionally been shown to be effective for the hydrometallation of alkenes and alkynes.

Considering that neutral group 14 element(iv) hydrides (*e.g.* R_3_EH, E = Si, Ge or Sn) do not possess any vacant valence orbitals, it is not surprising that they are poorly effective for the hydroelementation of alkenes and alkynes, at least in their own right. However, reactions of this type (particularly hydrosilylations) are of considerable synthetic importance, and can proceed, for example, in the presence of transition metal catalysts or radical initiators; and/or when subjected to UV irradiation or elevated temperatures.^6^,^7^

**Scheme 1:**
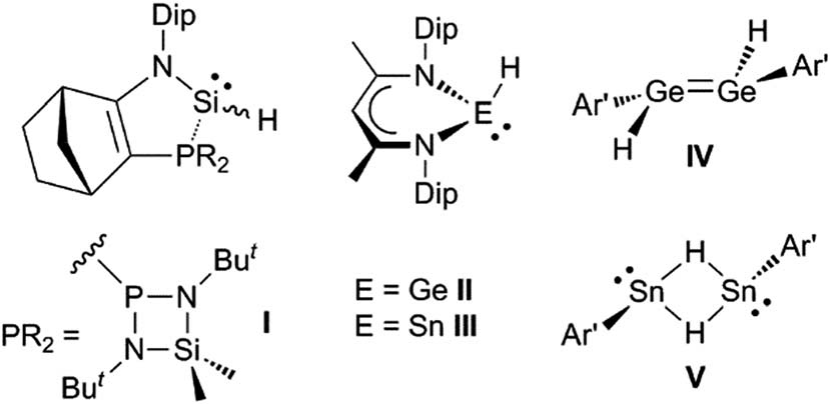
Examples of previously reported group 14 element(ii) hydride complexes (Dip = C_6_H_3_Pr^i^_2_-2,6; Ar′ = C_6_H_3_(C_6_H_3_Pr^i^_2_-2,6)_2_-2,6).

It would be a significant advantage if the addition of group 14 element-hydrogen bonds to unsaturated hydrocarbons could be effected in the absence of catalysts or initiators, and in a facile manner under ambient conditions. The first hints that this might be possible came with the kinetic stabilisation of group 14 element(ii) hydride complexes, a small number of which (*e.g.***I–V**, Scheme 1)^8^–^11^ have been reported since the turn of the millennium.^12^ Of these, the three-coordinate silicon(ii) hydride, **I**, has been shown to hydrosilylate cyclopentene and a series of terminal olefins at elevated temperatures (70–120 °C) and in the presence of large excesses of the alkene substrate.^8^ The latter reactions give rise to mixtures of regioisomers, in which the *anti*-Markovnikov product predominates. In one case, *i.e.* the reaction with trimethylsilylethylene, the reaction proceeds *via* an isolated [2 + 1] cycloadduct, *viz.* the silirane [**I**{η^2^-H_2_C=C(H)(SiMe_3_)}], which exists in equilibrium with **I** and free H_2_C=C(H)(SiMe_3_) at ambient temperature. With respect to hydrogermylation and hydrostannylation reactions, the three-coordinate species, **II** and **III**, have been shown to cleanly hydrometallate activated (ester substituted) terminal and internal alkynes at ambient temperature.^13^ Furthermore, the dimeric, three-coordinate metal(ii) hydride complexes, **IV** and **V**, react with *tert*-butylethylene at ambient temperature over 48 hours to give the alkyl/aryl substituted ditetrelenes [{Ar′E(CH_2_CH_2_Bu^*t*^)}_2_] (Ar′ = C_6_H_3_(C_6_H_3_Pr^i^_2_-2,6)_2_-2,6; E = Ge or Sn). Contrastingly, after 48 hours, the reaction of **IV** with excess cyclopentene at ambient temperature yielded only a mono-hydrogermylation product, *viz.* the hydrido-digermene, [Ar′(H)Ge=Ge(Cp)Ar′] (Cp = cyclopentyl).^14^ This suggests that the dissociation of **IV** to the two-coordinate hydrido-germylene, Ge(H)Ar′, in solution is minimal.

Recently, we have utilised extremely bulky amide ligands, developed in our group,^15^ to kinetically stabilise amido/hydrido-digermenes, *e.g.* [L^†^(H)Ge=Ge(H)L^†^] **1** (L^†^ = –N(Ar^†^) (SiPr^i^_3_); Ar^†^ = C_6_H_2_{C(H)Ph_2_}_2_Pr^i^-2,6,4),^16^ and isomeric dimeric hydrido-bridged stannylenes, *e.g.* [L^†^Sn(μ-H)_2_SnL^†^] **2**.^17^ These were shown to significantly dissociate to the unprecedented two-coordinate hydrido-tetrylenes,:E(H)(L^†^) (E = Ge **3** or Sn **4**), in hydrocarbon solutions. Subsequently, two-coordinate hydrido-germylenes, bearing even bulkier amide ligands, *e.g.* [:Ge(H)(L^OBut^)] (L^OBut^ = –N(Ar^†^){Si(OBu^*t*^)_3_}) were isolated in the solid state.^18^ Given that the six valence electron compounds **3** and **4** possess empty p-orbitals at their metal centres (*cf.* boranes) it was proposed that they would act as effective reagents for the hydrometallation of unsaturated substrates. This was shown to be the case for aldehydes and ketones, and, indeed, **3** and **4** were also shown to be highly efficient catalysts for the hydroboration of the same substrates.^19^ Here, we show that these hydrido-tetrylenes regiospecifically hydrometallate a variety of unactivated alkene and alkyne substrates at ambient temperature, and with unprecedented facility. In some cases, these hydrometallation reactions are shown to be cleanly reversible under ambient conditions. Preliminary further reactivity studies of the formed amido/alkyl-terylenes are also reported.

## Results and discussion

### (i) Hydrometallation of alkenes and alkynes

**Scheme 2:**
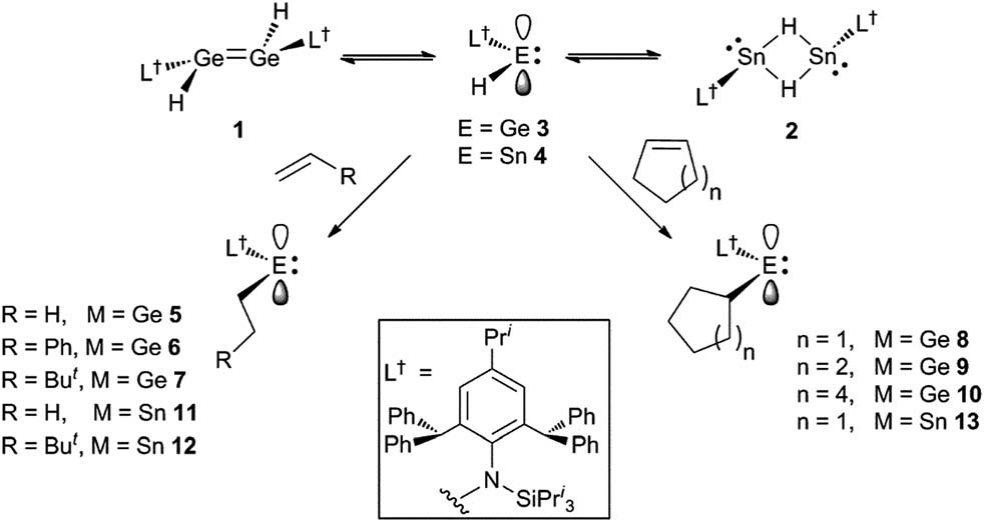
Synthesis of compounds **5–13**.

Treatment of toluene solutions of compound **3** (as an equilibrium mixture with **1**) with 1–1.5 equivalents of a range of unactivated terminal or cyclic alkenes, or 1 atm. of ethylene, led to almost instantaneous changes in the colour of the reaction solutions from orange to yellow at ambient temperature. This indicated that the hydrometallation reactions were complete in well under 1 minute. ^1^H NMR spectroscopic analyses of the reaction mixtures after *ca.* 10 min confirmed that the hydrometallation reactions were essentially quantitative after that time, affording the amido/alkyl germylenes, **5–10** (Scheme 2). These could be isolated as yellow crystalline solids in moderate to excellent yields upon work-up. Several corresponding reactions involving the tin(ii) hydride **4** (as an equilibrium mixture with **2**) were carried out at low temperature (–80 °C) due to the mild thermal instability of **4** at room temperature (solutions decompose over 2 days16*a*). Upon warming the reaction mixtures to *ca.* 20 °C, ^1^H NMR spectroscopic analyses revealed that hydrostannylation of the substrates had cleanly occurred to give **11–13**, which were isolated as crystalline solids in good yields (Scheme 2).

The facility of these uncatalysed olefin hydrogermylation and hydrostannylation reactions is unprecedented, and is likely a result of them preferentially involving the monomeric hydridoterylenes, **3** and **4**, over the dimeric species, **1** and **2** (*cf.* related carbonyl hydrometallations^19^). These coordinatively unsaturated species possess an empty p-orbital, which likely lowers the energy barrier to olefin hydrometallation, by allowing an interaction between the substrate and the group 14 metal center prior to the hydrometallation reaction (*cf.* alkene hydroelementations by boranes^2^ and the hydridosilylene **I**^8^). The fact that all of the alkene hydrometallation products reported here are monomeric, provides further evidence for the more active species in these reactions being the hydrido-tetrylenes, **3** and **4** (*cf.* dimeric products from alkene hydrometallations involving dimeric **IV** and **V**). It is also of note that the hydrometallations of all of the terminal alkenes regiospecifically yielded the *anti*-Markovnikov product, as is typically the case with alkene hydroborations.

**Fig. 1:**
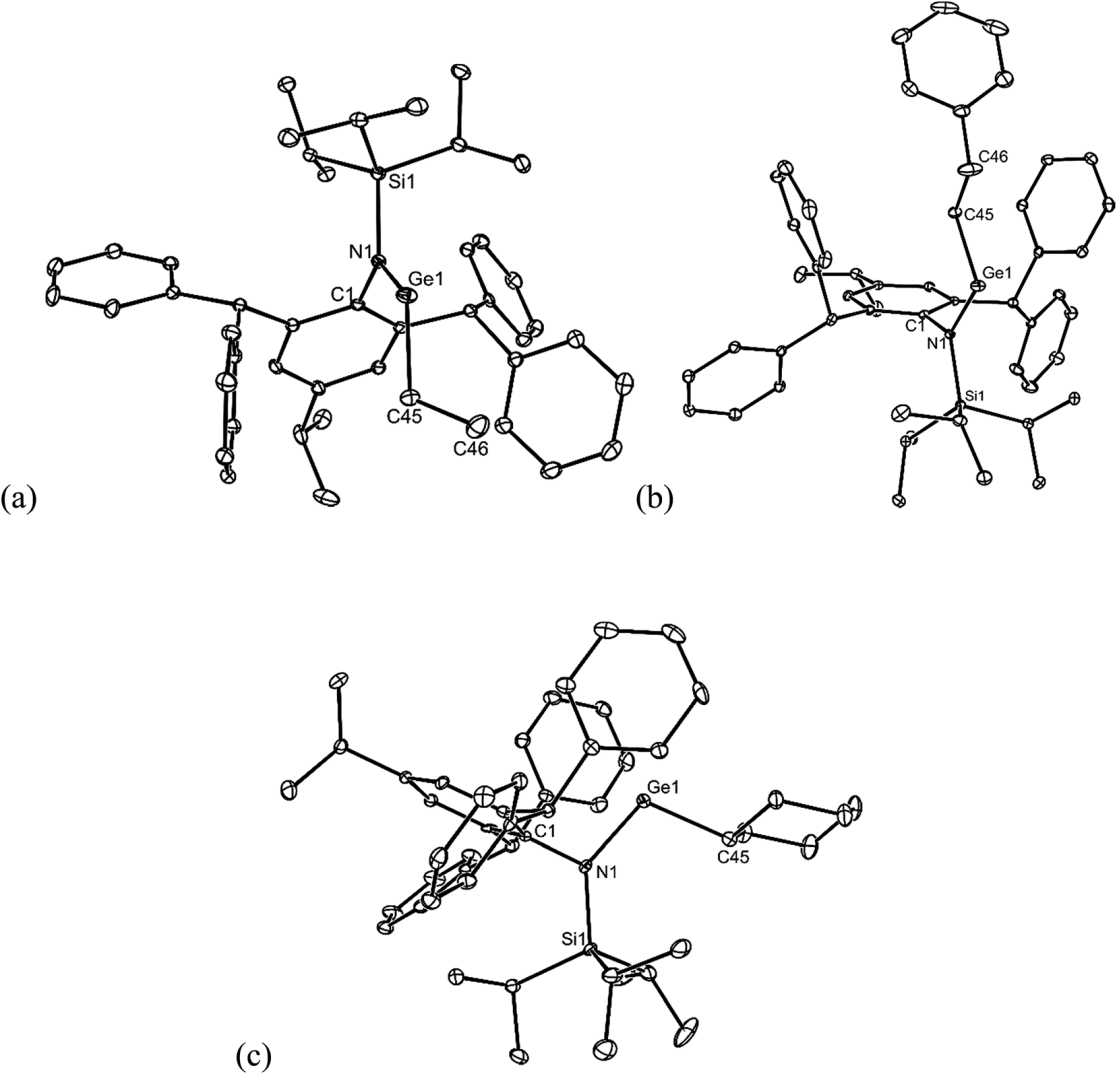
Molecular structures of (a) **5**, (b) **6**, and (c) **9** (25% thermal ellipsoids are shown; hydrogen atoms omitted). See Table 1 for selected metrical parameters.

All of the alkene hydrometallation products **5–13** are thermally stable in the solid state. The solution state NMR spectroscopic data for the compounds are fully consistent with their proposed monomeric structures, and do not warrant further comment here. X-ray crystallographic studies were used to confirm the monomeric nature of all compounds, which represent the first structurally characterised examples of two-coordinate amido/alkyl germylenes and stannylenes. Illustrative examples of the molecular structures of the compounds are depicted in Fig. 1 (see ESI† for the molecular structures of **8**, **10**, **11** and **13**), while selected geometrical parameters are collected in Table 1. It should be noted that, although the crystal structures of **7** and **12** confirmed the molecular connectivity of the compounds, they are not of a quality suitable for publication, and their geometrical parameters will not be discussed here.^20^ The geometries of all the structurally characterised compounds are similar, in that all of their N–E–C (E = Ge or Sn) angles are suggestive of stereochemically active lone pairs at the metal centre. Moreover, in each, the trigonal planar Si–N–C unit is close to co-planar with the C–E–N fragment. This potentially allows for overlap of the N p-orbital lone pair with an empty p-orbital at the E-centre, which would help prevent the already sterically bulky compounds from dimerising to ditetrelenes. There are no contacts between the Ge centres and any of the phenyl carbon atoms that would suggest significant Ge···aryl interactions in **5–10** (sum of van der Waals radii for Ge and C = 3.81 Å (ref. 21)). The closest Sn···C_phenyl_ contacts in **11–13** are considerably shorter, and are within the sum of van der Waals radii for Sn and C (3.87 Å(ref. 21)), which may indicate weak Sn···aryl interactions in those cases.

**Table 1:** Selected bond lengths (Å) and angles (°) for **5**, **6**, **8–11**, and **13** (E = Ge or Sn)

	**5**	**6**	**8**	**9**	**10**	**11**	**13**
E–N	1.874(2)	1.865(2)	1.885(3)	1.904(2)	1.898(2)	2.123(3)	2.127(4)
E–C	1.990(3)	1.991(2)	1.999(3)	2.031(4)	2.054(5)	2.182(4)	2.168(5)
N–E–C	105.4(1)	106.1(1)	110.6(1)	108.0(1)	109.8(3)	101.7(1)	102.8(2)
SiNEC (torsion)	5.4(1)	2.3(1)	10.7(1)	3.2(1)	1.3(1)	18.2(1)	13.5(3)
E···C_phenyl_^*a*^	3.461(3)	3.664(2)	3.257(3)	3.307(3)	3.330(3)	3.239(3)	3.173(6)

^*a*^The closest contact is given.

**Scheme 3:**
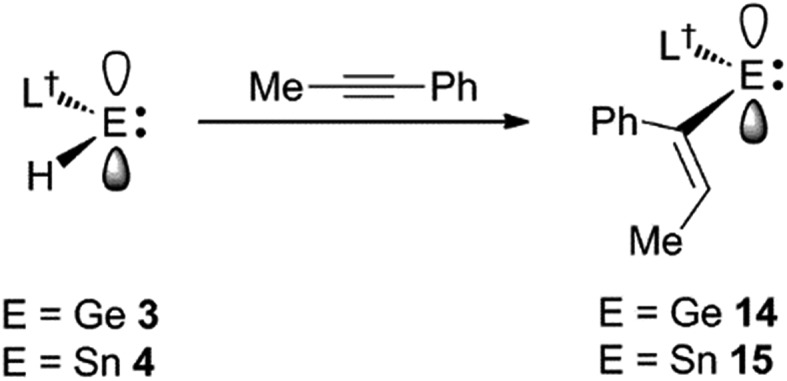
Synthesis of compounds **14** and **15**.

Considering the effectiveness of **3** and **4** as reagents for the hydrometallation of alkenes, it seemed likely that they would also be very reactive toward unactivated alkynes. To assess this, and to investigate if the metal hydrides could doubly hydrometallate alkynes, 1-phenyl-1-propyne was treated with two equivalents of either **3** or **4**. Analysis of the reaction mixtures indicated that only one hydrometallation event occurred in both cases, and within several minutes at ambient temperature. These reactions afforded compounds **14** and **15** respectively, in close to quantitative NMR spectroscopic yields, and moderate isolated yields. The reactions proceeded with complete regiospecificity, giving the *cis*-isomer with the L^†^E fragment bonded to the phenyl substituted alkenenic carbon (Scheme 3). It is likely that double hydrometallations do not occur in these reactions due to the imposing steric bulk of the L^†^E fragments. For sake of comparison, the hydroboration of 1-phenyl-1-propyne with boranes typically gives mixtures of regioisomers, the composition of which is dependent upon the borane employed.^22^

**Fig. 2:**
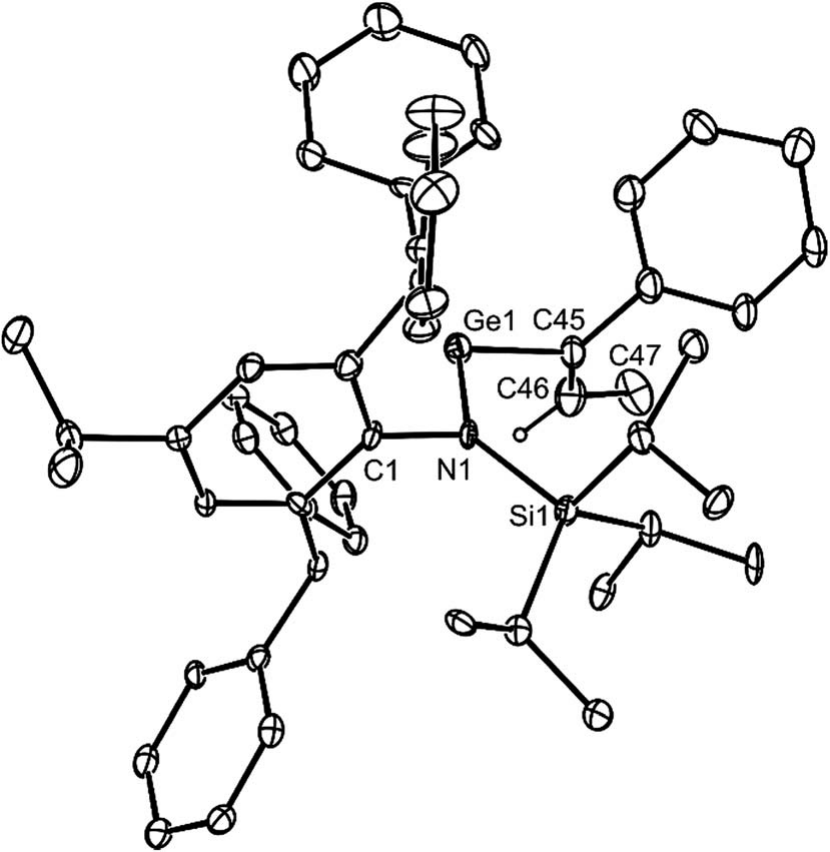
Molecular structure of **14** (25% thermal ellipsoids are shown; hydrogen atoms, except the alkenic proton, omitted). Selected bond lengths (Å) and angles (°) for **14**: Ge(1)–N(1) 1.881(6), Ge(1)–C(45) 1.99(1), C(45)–C(46) 1.35(1), closest Ge(1)···C_phenyl_ 3.27(1), N(1)–Ge(1)–C(45) 110.3(3). Selected bond lengths (Å) and angles (°) for **15**: Sn(1)–N(1) 2.122(5), Sn(1)–C(45) 2.229(8), C(45)–C(46) 1.32(1), closest Sn(1)···C_phenyl_ 3.26(1), N(1)–Sn(1)–C(45) 107.1(2).

Both **14** and **15** are thermally stable in the solid state and in solution. Their NMR spectra are in line with the proposed formulations of the compounds. The structures of the compounds were confirmed by X-ray crystallographic studies (see Fig. 2 for the molecular structure of **14**) which reveal both to be monomeric in the solid state with the alkeneic phenyl and methyl substituents *cis*- to one another. Their C(45)–C(46) distances reflect localised double bonds, while the geometries about the metal centres are similar to those in **5–13**. That is, the CNSi and NEC fragments are close to co-planar with one another, which allows for the possibility of N → E π-bonding in the compounds. In contrast, their C=C units are close to orthogonal to the NEC fragments, which discounts the possibility of any π-delocalisation over those fragments.

### (ii) Reversible alkene hydrometallations, and alkene isomerisations

**Scheme 4:**
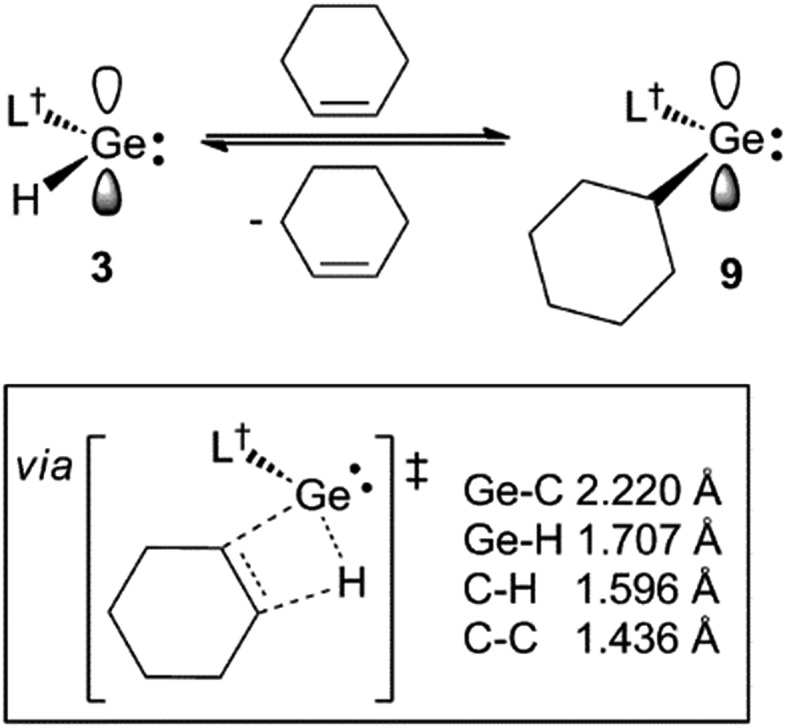
The reversible hydrogermylation of cyclohexene by **3**. A schematic representation of, and selected geometrical parameters for, the calculated (TPSS+D3(BJ)/def2-TZVPP) transition state for the reaction is shown in the box.

During characterisation of the cycloalkene hydrogermylation products **9** and **10**, it was noticed that ^1^H NMR spectra (C_6_D_6_) of pure samples of the compounds reproducibly exhibited signals due to the presence of small amounts of the germanium hydride equilibrium mixture, **1** and **3**, and the free cycloalkene. In addition, recrystallisation of the compounds, and **8**, repeatedly led to co-crystallisation with small amounts of **1**. Moreover, C_6_D_6_ solutions of the tin cyclopentyl compound, **13**, decomposed over several days at ambient temperature (to L^†^H, H_2_ and elemental tin), yet were stable for extended periods, even at 80 °C, in the presence of excess cyclopentene. All of these observations point to the hydrometallation products from the reactions of **3** and **4** with cycloalkenes being in equilibria with significant amounts of those reactants at room temperature (see Scheme 4 for the equilibrium between **3** and **9**). The net decomposition of **13** can be explained by the mild instability of the tin hydride **4** at ambient temperature, which upon decomposition, inextricably leads to loss of **13** from the equilibrium mixture in that case.

**Fig. 3:**
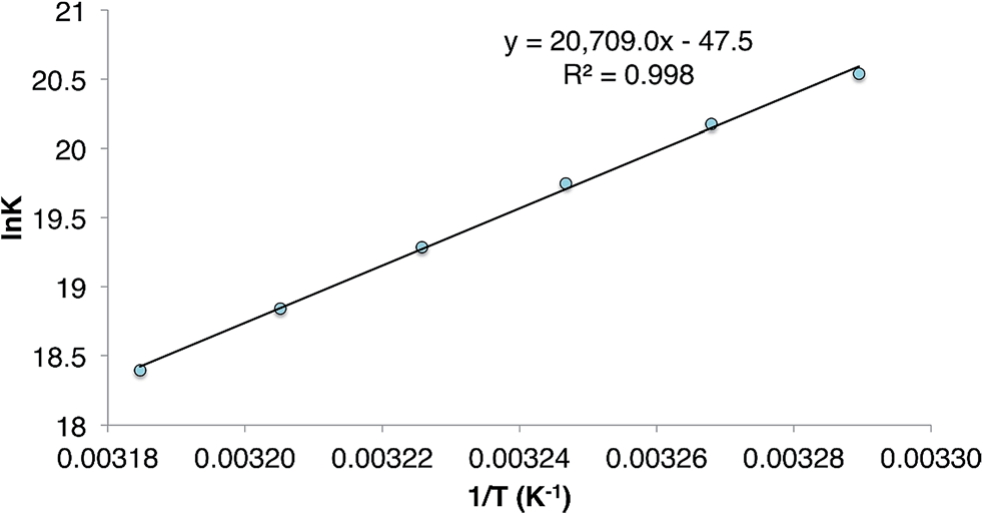
Plot of ln *K versus* 1/*T* for the equilibrium between **3** and **9**.

The reversibility of the reaction that gave **9** was explored by a VT ^1^H NMR spectroscopic study of a sample of the compound which was prepared by reaction of CyMgBr (Cy = cyclohexyl) with L^†^GeCl. This ensured the absence of any **1** in the purified sample of **9** used for the experiment. Despite this, dissolution of the compound in C_6_D_6_ again revealed the presence of a small amount (*ca.* 5% as determined by ^1^H NMR spectroscopy) of the **1**/**3** equilibrium mixture, and cyclohexene, as determined by a ^1^H NMR spectrum acquired at 20 °C. Heating the solution to 60 °C led to an increase in the quantities of these starting materials, which decreased when the solution was again cooled back to 20 °C. A van't Hoff analysis of this reversible process over the temperature range 304–314 K (see Fig. 3) revealed the forward hydrogermylation reaction to be exothermic (Δ*H*° = –172 kJ mol^–1^) with a relatively large entropic factor (Δ*S*° = 395 J mol^–1^). Accordingly, the Gibb's free energy for the exergonic hydrogermylation reaction at 298 K is fairly small (Δ*G* = –54 kJ mol^–1^), and therefore the weakly endergonic reverse reaction might be expected to be become more pronounced at elevated temperatures.

It is possible that the observed reverse reactions are due to β-hydride elimination processes, which are enabled by the coordinatively unsaturated nature of the two-coordinate metal centres in **8–10** and **13**. Indeed, inspection of the crystal structures of the compounds revealed, in each case, that the distance between the metal centre and the closest cycloalkyl β-hydrogen atom (range: 2.62–2.99 Å) is significantly less than the sum of the van der Waal's radii for E and H (E = Ge 3.21 Å, Sn 3.28 Å (ref. 21)). As far as we are aware, β-hydride elimination process involving germanium or tin alkyls, which are facile and reversible at room temperature, are unknown.^23^ With that said, there is one report of the hydrogermylation of a phosphaalkyne, P≡CBu^*t*^, by a three-coordinate germanium(ii) hydride, [(^Mes^Nacnac)GeH] (^Mes^Nacnac = [(MesNCMe)_2_CH]^–^, Mes = mesityl), which reversibly affords the phosphaalkenyl complex, [(^Mes^Nacnac)GeC(Bu^*t*^)=PH].^24^ The reversibility of this reaction at ambient temperature was proposed to involve migration of the phosphorus bound β-hydrogen to the germanium centre in [(^Mes^Nacnac)GeC(Bu^*t*^)=PH].

To explore the possibility of facile β-hydrogen elimination processes being the origin of the reversibility of the reactions that gave **8–10**, DFT calculations were carried out at several levels of theory on the hydrogermylation reaction that gave **9** (see ESI† for full details). These indicated that the reaction is exergonic by an amount (Δ*G* = –42.3 kJ mol^–1^ at M06-2X+D3/def2-TZVPP//TPSS+D3/def2-TZVPP) that is small, and not dissimilar to that found from the experimental van't Hoff analysis of the reaction. Importantly, the reverse reaction was, indeed, found to proceed *via* a β-hydride elimination process, involving a transition state with a four-membered GeC_2_H ring (see Scheme 4 and ESI†). In combination with the small free energy of the reaction, the relatively low free energy of activation for this transition state (Δ^‡^*G* = 76.6 kJ mol^–1^), is fully consistent with the experimentally observed equilibrium for the reaction that gave **9**.

**Scheme 5:**
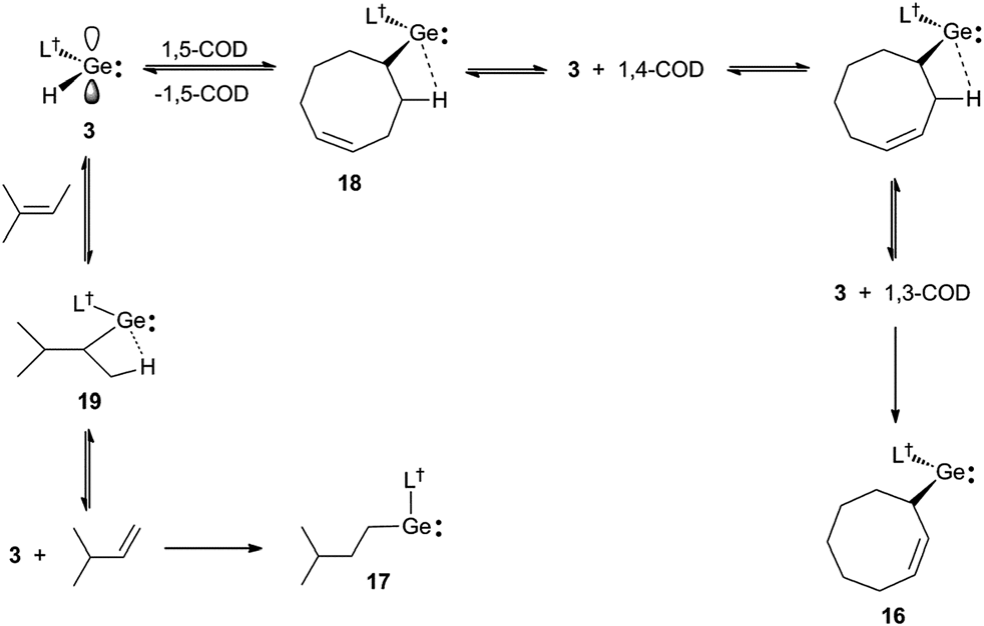
Synthesis of **16** and **17***via* hydrogermylation and isomerisation of 1,5-cyclooctadiene and 2-methyl-2-butene, respectively.

Further evidence that the reversibility of the cyclic alkene hydrogermylation reactions proceed *via* β-hydrogen elimination processes, comes from the reactions of **3** with 1,5-cyclooctadiene (1,5-COD) and 2-methyl-2-butene (Scheme 5). In both cases the expected hydrometallation products were not observed, and instead products, **16** and **17**, that apparently result from the hydrogermylation of isomerised alkenes, were isolated in moderate yields. It is worth mentioning that treatment of 1,5-cyclooctadiene with two equivalents of **3** did not lead to a double hydrogermylation product, and no reaction occurred between **3** and the tetra-substituted alkenes, R_2_C=CR_2_ (R = Me or Ph). These observations presumably result from the considerable steric bulk of the monomeric hydridogermylene, **3**.

It is possible that the formation of **16** proceeds *via* the expected hydrogermylation product, **18**, as an intermediate. This then undergoes a β-hydrogen elimination to give **3** and 1,4-cyclooctadiene (1,4-COD). Hydrogermylation of this by **3**, and another β-hydrogen elimination event, yields **3** and 1,3-cyclooctadiene (1,3-COD), the latter of which is then hydrogermylated to give the observed product, **16**. Similarly, hydrogermylation of 2-methyl-2-butene affords the initially expected product, **19**, which β-hydride eliminates to give **3** and 3-methyl-1-butene. Hydrogermylation of this olefin then leads to the observed product, **17**. The facility of these reactions highlights the potential that **3**, and related reagents, have for the selective stoichiometric isomerisation of alkenes. While such isomerisations are common for transition metal systems,^25^ they are rare for main group compounds.

**Fig. 4:**
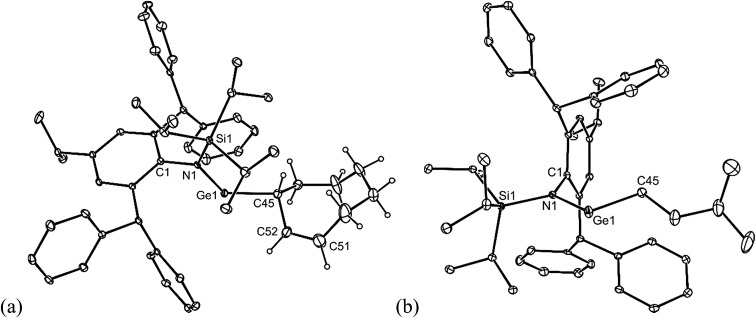
Molecular structures of (a) **16** and (b) **17** (25% thermal ellipsoids are shown; hydrogen atoms, except cyclooctenyl protons, omitted). Selected bond lengths (Å) and angles (°) for **16**: Ge(1)–N(1) 1.890(2), Ge(1)–C(45) 2.034(3), C(51)–C(52) 1.372(6), N(1)–Ge(1)–C(45) 108.70(11). Selected bond lengths (Å) and angles (°) for **17**: Ge(1)–N(1) 1.872(2), Ge(1)–C(45) 1.988(3), N(1)–Ge(1)–C(45) 105.57(10).

Both **16** and **17** were crystallographically characterised, and their molecular structures are depicted in Fig. 4. These show them to be monomeric with geometries that are comparable to those of the other amido/alkyl germylenes, **5–10**, reported here. In the case of **16**, the presence and location of the residual double bond of its cyclooctenyl moiety is confirmed by the shortness of the C(51)–C(52) linkage (1.372(6) Å).

### (iii) Reactivity studies of amido/alkyl germylenes and stannylenes

**Scheme 6:**
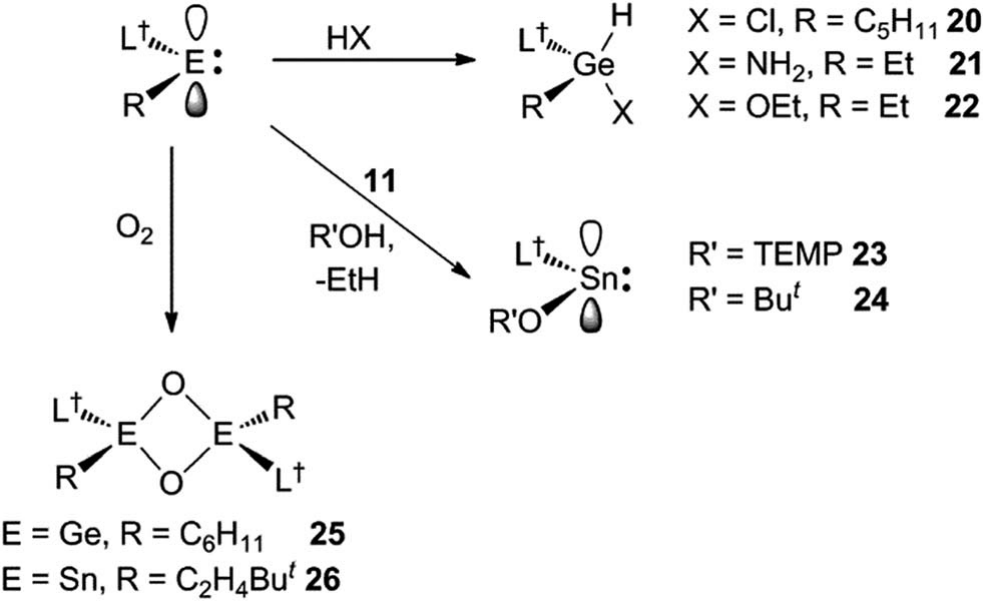
Synthesis of compounds **20–26**.

Preliminary further reactivity studies were carried out on examples of the amido/alkyl germylene and stannylene complexes prepared here, with a view to utilising these compounds in catalytic synthetic protocols. Initially, the oxidative addition (OA) of H_2_ and hydridic reagents (*e.g.* HBpin, HBcat, PhSiH_3_, Et_3_SiH, (EtO)_3_SiH and DIBAL) to **5–13** was explored, but in no case was a reaction observed under ambient conditions. Attention then turned to the reaction of stoichiometric amounts of protic reagents with **5**, **8** and **11**. In the case of the germylenes, the oxidative addition of HCl, NH_3_ or EtOH to the Ge centres of **5** or **8** occurred, to give a few crystals of **20** (amongst several other unidentified products), and good isolated yields of **21** and **22**, respectively (Scheme 6). Solutions of **21** and **22** are resistant to reductive elimination (RE) of ethane or cyclopentane, even when heated to 50 °C for one hour. It is noteworthy that the reactions that gave **20–22** are comparable to related oxidative additions of HF,^26^ NH_3_^27^ and EtOH^28^ to germylenes, that have appeared in the literature.

Different outcomes resulted from the reactions of the stannylene **11** with protic reagents. These were not clean, and typically generated product mixtures that contained significant amounts of the secondary amine, L^†^H. The two exceptions here were the reactions with stoichiometric amounts of the bulkier reagents TEMPOH (TEMP = 2,2,6,6-tetramethylpiperidinyl) and Bu^*t*^OH. These afforded moderate to good isolated yields of the piperidinyl N-oxide product, **23**, and the known tin *tert*-butoxide, **24**,^19^ respectively (Scheme 6). When these reactions were followed by ^1^H NMR spectroscopy, the generation of significant amounts of ethane (and smaller quantities of L^†^H) was observed. It cannot be sure if these reactions proceed *via* initial oxidative additions of the O–H bond of the reagents to the Sn^II^ center of **11**, prior to reductive elimination of ethane, but given the formation of the stable germanium(iv) ethoxide, **22**, this is certainly a possibility (*cf.* related “OA/RE” reactions of H_2_ and NH_3_ with Ar′_2_Sn: (ref. 27)).

In attempts to form a rare example of a three-coordinate germanone (R_2_Ge=O),^29^ and the first example of a corresponding stannanone (R_2_Sn=O), toluene solutions of the germylene, **9**, and stannylene, **12**, were reacted with excess O_2_. Instead of yielding monomeric products, the dimeric oxo-bridged species, **25** and **26**, were obtained in moderate isolated yields (Scheme 6). It is apparent that the steric shielding of the metal centres in the compounds is not sufficient to prevent dimerisation of the target heavier ketone products. In this respect, there is ample literature precedent for the oxidation of germylenes and stannylenes to give dimeric systems, comparable to **25** and **26**.^30^,^31^

**Fig. 5:**
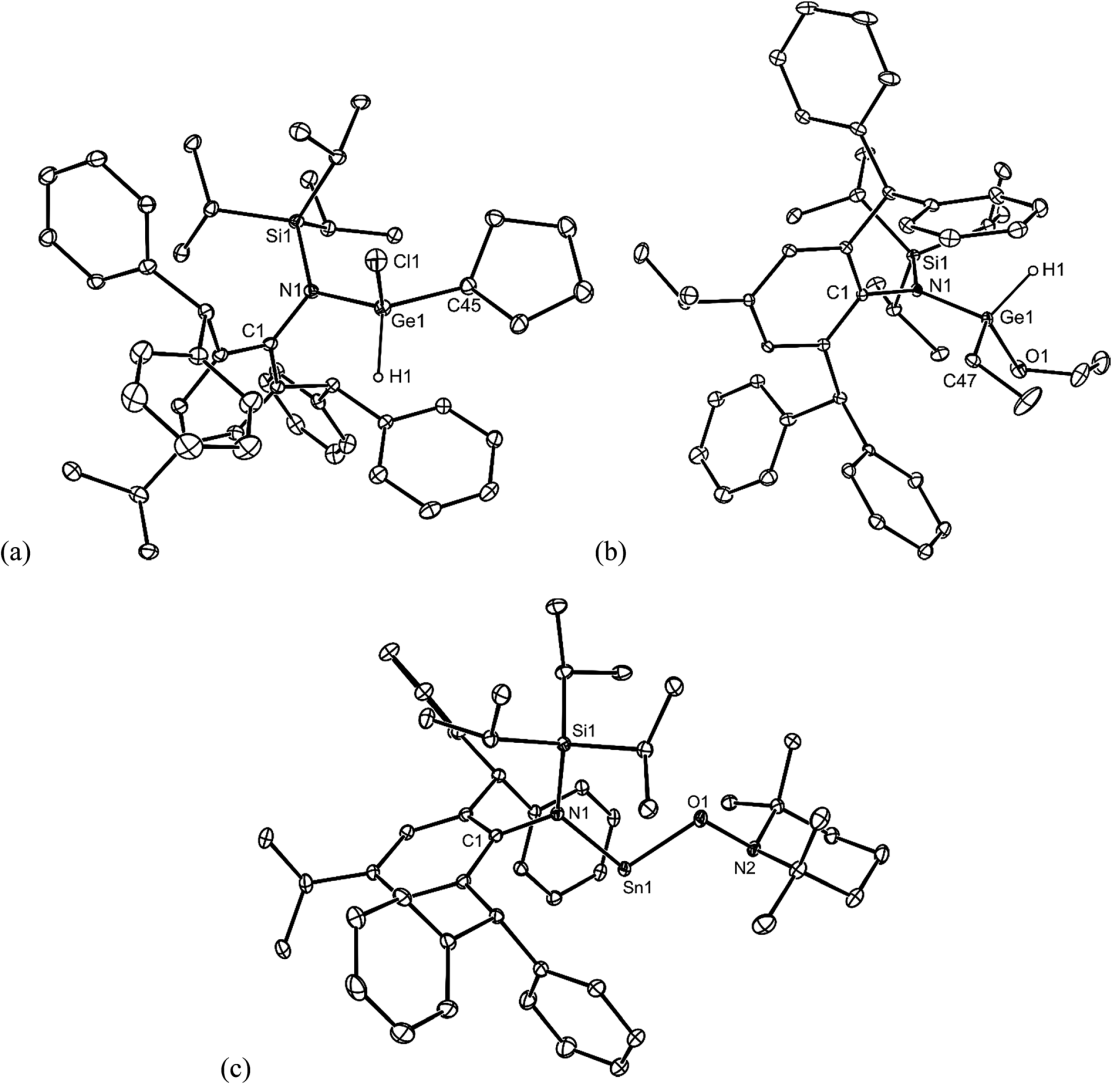
Molecular structures of (a) **20**, (b) **22**, and (c) **23** (25% thermal ellipsoids are shown; hydrogen atoms, except hydrides, omitted). Selected bond lengths (Å) and angles (°) for **20**: Ge(1)–N(1) 1.864(3), Ge(1)–C(45) 1.952(4), Ge(1)–Cl(1) 2.189(1), Ge(1)–H(1) 1.53(2), N(1)–Ge(1)–H(1) 108(2), C(45)–Ge(1)–Cl(1) 103.1(1). Selected bond lengths (Å) and angles (°) for **22**: Ge(1)–O(1) 1.805(2), Ge(1)–N(1) 1.860(2), Ge(1)–C(47) 1.930(3), Ge(1)–H(1) 1.47(3), N(1)–Ge(1)–H(1) 109(2), O(1)–Ge(1)–C(47) 112.0(2). Selected bond lengths (Å) and angles (°) for **23**: Sn(1)–O(1) 2.0393(16), Sn(1)–N(1) 2.1282(18), O(1)–N(2) 1.463(2), O(1)–Sn(1)–N(1) 96.76(7), N(2)–O(1)–Sn(1) 108.21(12).

**Fig. 6:**
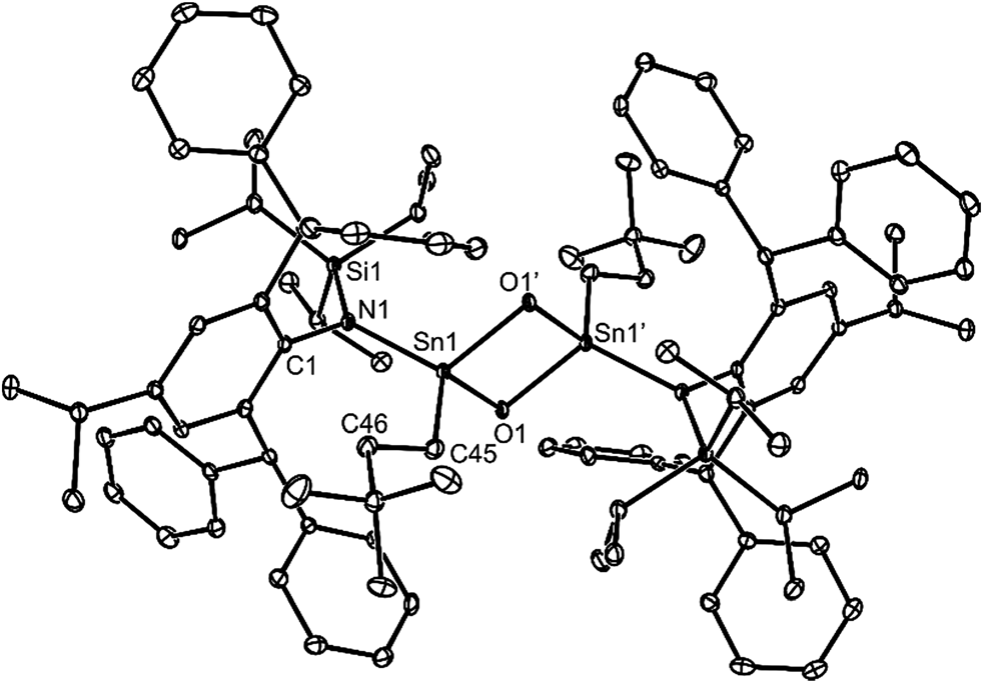
Molecular structure of **26** (25% thermal ellipsoids are shown; hydrogen atoms omitted). Selected bond lengths (Å) and angles (°): Sn(1)–O(1) 2.001(3), Sn(1)–O(1)′ 2.012(2), Sn(1)–N(1) 2.056(3), Sn(1)–C(45) 2.140(4), N(1)–Sn(1)–C(45) 115.91(14), O(1)–Sn(1)-O(1)′ 83.63(11), Sn(1)–O(1)–Sn(1)′ 96.37(11).

No spectroscopic data could be obtained for the HCl oxidative addition product, **20**, due to the very low yield of the compound. The NMR spectroscopic data for the other products, **21–23**, are as would be expected, though it is worthy of mention that the chiral Ge centres in **21** and **22** give rise to two multiplet resonances for the diastereotopic CH_2_ units of both the ethyl and ethoxide ligands. The X-ray crystal structures of **20**, **22** and **23** were determined and their molecular structures are shown in Fig. 5. A preliminary crystal structure of the NH_3_ addition product, **21**, was also obtained, though this was not of publishable quality. Despite this, the molecular connectivity in the compound is unambiguous, and as proposed. The hydride ligands of **20** and **22** were located from difference maps, and refined isotropically, which revealed the chiral Ge centres of those compounds to have distorted tetrahedral geometries. In contrast, the tin centre of **23** is two-coordinate, and the SiNC fragment is essentially co-planar with the NSnON unit, which allows for the possibility of N→Sn π-bonding in the compound. It is worthy of mention that there is only one other structurally characterised tin piperidinyl N-oxide complex reported in the literature, [{CH_2_C(SiMe_3_)_2_}_2_Sn(OTEMP)_2_],^32^ though the metal centre in this is in the +4 oxidation state. The dimeric nature of **25** and **26** was confirmed by X-ray crystallographic studies (see Fig. 6 for the molecular structure of **26**), which also showed their oxide ligands to essentially symmetrically bridge two distorted tetrahedral metal centres.

## Conclusion

In summary, reactions of solution stable two-coordinate hydrido-tetrylenes with a variety of unactivated cyclic and acyclic alkenes, and one internal alkyne, lead to the unprecedentedly rapid and regioselective hydrometallation of the unsaturated substrate at ambient temperature. The products of the alkene hydrometallations represent the first structurally characterised examples of two-coordinate amido/alkyl germylenes and stannylenes. In the cases of the cycloalkene hydrometallations, the reactions were shown to be cleanly reversible under ambient conditions. The results of computational and experimental van't Hoff analyses of one such reaction, strongly suggest that its reversal proceeds *via* β-hydride elimination from the cycloalkyl ligand, regenerating the cycloalkene and hydrido-tetrylene starting materials. Further evidence for this proposal comes from the reactions of a hydrido-germylene with 1,5-cyclooctadiene and 2-methyl-2-butene, both of which seemingly proceed *via* intermediate β-hydride elimination processes, and the clean isomerisation of the alkene involved, prior to its ultimate hydrogermylation. In addition, the element-hydrogen bonds of several protic compounds have been shown to oxidatively add to the germanium(ii) centre of two of the amido/alkyl germylenes prepared in this study, while similar reactions with an equivalent stannylene proceed *via* alkane elimination, and generation of tin(ii) products. Oxidations of two amido/alkyl tetrylenes with O_2_ have been shown to give four-coordinate, oxo-bridged metal(iv) dimers. We continue to explore the stabilisation and synthetic utility of low oxidation state group 14 element compounds.

## Supplementary Material

Supplementary information

Crystal structure data
