# Supplementary material for: Two-coordinate group 14 element(ii) hydrides as reagents for the facile, and sometimes reversible, hydrogermylation/hydrostannylation of unactivated alkenes and alkynes
Source: Chem Sci. 2015 Sep 22;6(12):7249–57. doi: 10.1039/c5sc03376d (PMC5950753; doi:10.1039/c5sc03376d)
Supplement: Supplementary file 1 [file SC-006-C5SC03376D-s001.pdf]

Electronic Supplementary Material (ESI) for Chemical Science.
This journal is © The Royal Society of Chemistry 2015

Two-Coordinate Group 14 Element(II) Hydrides as Reagents for the Facile, and Sometimes Reversible, Hydrogermylation/Hydrostannylation of Unactivated Alkenes and Alkynes

Terrance J. Hadlington,[a] Markus Hermann,[b] Gernot Frenking,*,[b] and Cameron Jones*,[a]

[a] School of Chemistry, PO Box 23, Monash University, VIC, 3800, Australia.

[b] Fachbereich Chemie, Philipps-Universität Marburg, 35032, Marburg, Germany.

Supplementary Information (30 pages)

Contents

**1. Experimental**	**S2**
**2. X-Ray Crystallography**	**S14**
**3. Computational Studies**	**S23**
**4. References**	**S29**

1. Experimental

General considerations.

All manipulations were carried out using standard Schlenk and glove box techniques under an atmosphere of high purity dinitrogen. Diethyl ether was distilled over Na/K alloy (25:75), while THF, hexane and toluene were distilled over molten potassium. ^{1}H, ^{13}C{^{1}H}, ^{29}Si{^{1}H} and ^{119}Sn{^{1}H} NMR spectra were recorded on either Bruker DPX300, Bruker AvanceIII 400 or Varian Inova 500 spectrometers and were referenced to the resonances of the solvent used, external $SiMe_4$, or external $SnMe_4$. Where ^{119}Sn{^{1}H} NMR data are not reported for tin containing compounds, the relevant spectra were acquired, though no signals were observed. Mass spectra were recorded on an Agilent Technologies 5975D inert MSD with a solid state probe. IR spectra were recorded on solid samples using a Agilent Cary 630 attenuated total reflectance (ATR) spectrometer. Melting points were determined in sealed glass capillaries under dinitrogen, and are uncorrected. Microanalyses were carried out at the Science Centre, London Metropolitan University. The compounds **1**,[1] **2**,[2] [L†GeCl],[1] [L†SnOBu^{t}][3] and dry TEMPOH[4] were prepared by literature procedures. High purity ethylene and ammonia (99.9%) were used as received, while O_2 was dried over P_2O_5 prior to use. All other reagents were used as received.

General procedure for the synthesis of amido/alkyl and amido/alkenyl substituted germylenes: To a stirred solution of **1** (in equilibrium with **3**) in toluene at ambient temperature was added the neat alkene or alkyne substrate (typically 2.6 equiv.), or the solution was put under an atmosphere of ethylene (1 atm.). The colour of the reaction mixtures rapidly changed from bright orange to yellow. The mixtures were stirred for a further 1 h, after which time all volatiles were removed under vacuum. The residues were extracted into warm hexane and filtered. The filtrates were concentrated to the point of incipient crystallisation, then placed at -30 °C or 4 °C until the compound had crystallised. N.B. For the cyclic alkyl substituted species, **8-10**, *ca.* 5 equivs of the relevant alkene was added to the filtrate, to hinder equilibration with **3**, which can lead to co-crystallisation with dimeric **1**.

General procedure for the synthesis of amido/alkyl and amido/alkenyl substituted stannylenes: These compounds were synthesised by two routes:

Route (a): The relevant alkene or alkyne substrate (3 equiv. for terminal alkenes and 1-phenyl-1-propyne, or 12 equiv. for cyclic alkenes) was added to a solution of **2** (in equilibrium with **4**) in toluene at -80 ºC. The reaction mixtures were then warmed to ambient temperature, and stirred for a further 1 h. Subsequently, volatiles were removed under vacuum, the residues extracted into

hexane, and the extract filtered. The filtrates were concentrated to the point of incipient crystallisation, then placed at -30 °C or 4 °C until the compound had crystallised.

Route (b): Neat HBpin was added to a solution of [L†SnOBu^t] in toluene at -30 ºC, followed by the relevant substrate (*ca.*1.5 equiv. for terminal alkenes and 1-phenyl-1-propyne, or *ca.* 6 equiv. for cyclic alkenes). The reaction mixtures were warmed to ambient temperature, resulting in deep yellow/brown solutions. Subsequently, volatiles were removed under vacuum, the residues extracted into hexane, and the extract filtered. The filtrates were concentrated to the point of incipient crystallisation, then placed at -30 °C or 4 °C until the compound had crystallised.

N.B. For both methods, the cyclic alkyl substituted species, **13**, was recrystallised with an excess of cyclopentene added to the filtrate, in order to hinder equilibration with **4**, and thus avoiding decomposition of **13** to L†H, H_2 and tin metal.

Preparation of [L†GeEt] 5. Using **1** (0.30 g, 0.22 mmol), and ethylene (1 atm.). The product was recrystallised from a solution in 5 mL hexane at 4 ºC (215 mg, 68 %).

Alternative synthesis. To a suspension of flame-dried fine Mg turnings (43 mg, 1.77 mmol) in diethyl ether (15 mL) was added EtBr (112 µL, 1.50 mmol), and the reaction mixture subsequently heated at reflux for 4 h. The resultant suspension was then cooled to ambient temperature, and filtered into a solution of [L†GeCl] (1.0 g, 1.38 mmol) in THF (40 mL) at -80 ºC. The mixture was then warmed to ambient temperature, and stirred for a further 30 min. Over this time the reaction solution became yellow. An ^{1}H NMR spectroscopic analysis showed essentially quantitative conversion to the desired [L†GeEt] by this time. All volatiles were subsequently removed *in vacuo*, and the residue extracted in hot hexane, followed by filtration. Removal of volatiles from the filtrate *in vacuo* resulted in **5** as a free-flowing, analytically pure micro-crystalline yellow powder (800 mg, 80 %). M.p. 92-100 °C (melt); ^{1}H NMR (C_6D_6, 400 MHz, 298 K): δ = -1.02 (br m, 2H, GeC*H*$_2$), 0.55 (t, $^3J_{HH}$ = 7.2 Hz, 3H, GeCH$_2$C*H*$_3$), 1.04 (d, $^3J_{HH}$ = 6.8 Hz, 6H, Ar†-*p*-CH(C*H*$_3$)$_2$), 1.27 (d, $^3J_{HH}$ = 7.6 Hz, 18H, SiCH(C*H*$_3$)$_2$), 1.77 (sept, $^3J_{HH}$ = 7.6 Hz, 3H, SiC*H*(CH$_3$)$_2$), 2.60 (sept, $^3J_{HH}$ = 6.8 Hz, 1H, Ar†-*p*-C*H*(CH$_3$)$_2$), 6.17 (s, 2H, C*H*Ph$_2$), 7.07-7.41 (m, 22H, Ar-*H*); ^{13}C{^{1}H} NMR (C_6D_6, 75.5 MHz, 298 K): δ = 8.0 (GeCH$_2$*C*H$_3$), 14.9 (Si*C*H(CH$_3$)$_2$), 20.3 (SiCH(*C*H$_3$)$_2$), 24.6 (Ar†-*p*-CH(*C*H$_3$)$_2$), 34.2 (Ar†-*p*-*C*H(CH$_3$)$_2$), 37.2 (Ge*C*H$_2$CH$_3$), 51.9 (*C*HPh$_2$), 126.9, 127.7, 128.7, 129.5, 130.0, 130.3, 138.5, 143.3, 145.6, 145.9, 146.7, 149.4 (Ar-*C*); ^{29}Si{^{1}H} NMR (C_6D_6, 80 MHz, 298 K): δ = 10.4; IR, ν/cm^{-1} (ATR): 1597 (m), 1379 (m), 1226 (m), 1201 (m), 1118 (s), 1031 (s), 882 (s), 816 (s), 659 (s); MS/EI *m/z* (%): 696.3 (M$^+$-C_2H_5, 80), 580.3 (L$^{†+}$-Pr^i, 100); anal. calcd. for $C_{46}H_{57}GeNSi$: C, 76.24 %; H, 7.93 %; N, 1.93 %; found: C, 76.60 %; H, 8.04 %; N, 2.02 %.

Preparation of [L†Ge(C_2H_4Ph)] 6. Using **1** (0.20 g, 0.14 mmol), and styrene (0.34 mmol, 40 μL). The yellow product was recrystallised from 10 mL hexane at 4 ºC (115 mg, 51 %). ^{1}H NMR (C_6D_6, 400 MHz, 298 K): δ = -0.60 (br, 2H, GeC*H*$_2$), 1.02 (d, $^3J_{HH}$ = 6.8 Hz, 6H, Ar†-*p*-CH(C*H*$_3$)$_2$), 1.26 (d, $^3J_{HH}$ = 7.6 Hz, 18H, SiCH(C*H*$_3$)$_2$), 1.76 (sept, $^3J_{HH}$ = 7.6 Hz, 3H, SiC*H*(CH$_3$)$_2$), 2.15 (m, 2H, GeCH$_2$C*H*$_2$), 2.57 (sept, $^3J_{HH}$ = 6.8 Hz, 1H, Ar†-*p*-C*H*(CH$_3$)$_2$), 6.16 (s, 2H, C*H*Ph$_2$), 6.73-7.40 (m, 27H, Ar-*H*); ^{13}C{^{1}H} NMR (C_6D_6, 75.5 MHz, 298 K): δ = 14.3 (Si*C*H(CH$_3$)$_2$), 19.7 (SiCH(*C*H$_3$)$_2$), 24.0 (Ar†-*p*-CH(*C*H$_3$)$_2$), 29.7 (Ge*C*H$_2$), 33.6 (Ar†-*p*-*C*H(CH$_3$)$_2$), 46.8 (GeCH$_2$*C*H$_2$), 52.3 (*C*HPh$_2$), 125.4, 126.4, 127.4, 128.2, 128.8, 129.0, 129.6, 129.7, 129.9, 130.2, 137.9, 142.9, 145.1, 145.2, 146.1, 148.8 (Ar-*C*); ^{29}Si{^{1}H} NMR (C_6D_6, 80 MHz, 298 K): δ = 10.7; IR, ν/cm^{-1} (ATR): 1597 (m), 1379 (m), 1258 (m), 1113 (m), 1031 (s), 877 (s), 809 (s), 744 (s), 661 (s); MS/EI *m/z* (%): 696.4 (M$^+$-(CH$_2$)$_2$Ph, 1), 623.4 (L$^{†+}$, 55), 580.4 (L$^{†+}$-Pr^i, 100); anal. calcd. for $C_{52}H_{61}$GeNSi: C, 78.00 %; H, 7.68 %; N, 1.75 %; found: C, 77.90 %; H, 7.75 %; N, 1.82 %.

Preparation of [L†Ge(C_2H_4Bu^t)] 7. Using **1** (0.20 g, 0.14 mmol), and 3,3-dimethyl-1-butene (0.36 mmol, 46 μL). The yellow product was recrystallised from 5 mL hexane at 4 ºC (90 mg, 38 %); M.p. 122-128 °C (melt); ^{1}H NMR (C_6D_6, 500 MHz, 298 K): δ = -0.72 (br, 2H, GeC*H*$_2$), 0.48 (s, 9H, C(C*H*$_3$)$_3$), 0.86 (m, 2H, GeCH$_2$C*H*$_2$), 1.05 (d, $^3J_{HH}$ = 6.8 Hz, 6H, Ar†-*p*-CH(C*H*$_3$)$_2$), 1.24 (d, $^3J_{HH}$ = 7.6 Hz, 18H, SiCH(C*H*$_3$)$_2$), 1.67 (sept, $^3J_{HH}$ = 7.6 Hz, 3H, SiC*H*(CH$_3$)$_2$), 2.62 (sept, $^3J_{HH}$ = 6.8 Hz, 1H, Ar†-*p*-C*H*(CH$_3$)$_2$), 6.18 (s, 2H, C*H*Ph$_2$), 6.95-7.48 (m, 22H, Ar-*H*); ^{13}C{^{1}H} NMR (C_6D_6, 75.5 MHz, 298 K): δ = 14.3 (Si*C*H(CH$_3$)$_2$), 19.7 (SiCH(*C*H$_3$)$_2$), 24.1 (Ar†-*p*-CH(*C*H$_3$)$_2$), 29.2 (C(*C*H$_3$)$_3$), 31.5 (Ar†-*p*-*C*H(CH$_3$)$_2$), 33.8 (GeCH$_2$*C*H$_2$), 36.5 (Ge*C*H$_2$), 41.8 (*C*(CH$_3$)$_3$), 51.0 (*C*HPh$_2$), 126.3, 127.1, 129.5, 130.0, 130.1, 137.4, 140.8, 142.7, 145.0, 145.9, 146.2, 149.1 (Ar-*C*); ^{29}Si{^{1}H} NMR (C_6D_6, 80 MHz, 298 K): δ = 10.5; IR, ν/cm^{-1} (ATR): 1597 (m), 1360 (m), 1231 (s), 1117 (m), 1031 (m), 879 (s), 808 (s), 729 (s), 655 (s); MS/EI *m/z* (%): 696.3 (L†Ge$^+$, 3), 623.4 (L$^{†+}$, 44), 580.3 (L$^{†+}$-Pr^i, 100); anal. calcd. for $C_{50}H_{65}$GeNSi: C, 76.92 %; H, 8.39 %; N, 1.79 %; found: C, 75.92 %; H, 7.43 %; N, 2.13 %.

Preparation of [L†Ge(C_5H_9)] 8. Using **1** (0.20 g, 0.14 mmol), and cyclopentene (0.36 mmol, 32 μL). The yellow product was rerystallised from 5 mL hexane, with additional cyclopentene (80 μL) added, at -30 ºC (190 mg, 73 %). M.p. 137-146 °C (melt); ^{1}H NMR (C_6D_6, 400 MHz, 298 K): δ = 0.92 (br m, 4H, Ge(C$_5$H$_9$)-C*H*$_2$), 1.01 (d, $^3J_{HH}$ = 6.8 Hz, 6H, Ar†-*p*-CH(C*H*$_3$)$_2$), 1.21 (br m, 1H, GeC*H*), 1.28 (d, $^3J_{HH}$ = 7.6 Hz, 18H, SiCH(C*H*$_3$)$_2$), 1.42 (br m, 4H, Ge(C$_5$H$_9$)-C*H*$_2$), 1.77 (sept, $^3J_{HH}$ = 7.6 Hz, 3H, SiC*H*(CH$_3$)$_2$), 2.57 (sept, $^3J_{HH}$ = 6.8 Hz, 1H, Ar†-*p*-C*H*(CH$_3$)$_2$), 6.27 (s, 2H, C*H*Ph$_2$), 6.96-7.38 (m, 22H, Ar-*H*); ^{13}C{^{1}H} NMR (C_6D_6, 75.5 MHz, 298 K): δ = 15.7 (Si*C*H(CH$_3$)$_2$), 20.0 (SiCH(*C*H$_3$)$_2$), 24.1 (Ar†-*p*-CH(*C*H$_3$)$_2$), 25.9 (Ge(C$_5$H$_9$)-*C*H$_2$), 27.4 (Ge(C$_5$H$_9$)-*C*H$_2$), 33.7 (Ar†-*p*-

CH(CH_3)$_2$), 50.2 (GeCH), 52.3 (CHPh$_2$), 126.5, 127.1, 128.5, 128.7, 129.0, 130.2, 130.8, 141.5, 143.4, 144.4, 145.7, 147.8 (Ar-C); ^{29}Si{^{1}H} NMR (C_6D_6, 80 MHz, 298 K): δ = 7.4; IR, ν/cm^{-1} (ATR): 1596 (w), 1190 (m), 1069 (m), 1001 (m), 881 (s), 761(s), 714 (s), 646 (s); MS/EI *m/z* (%): 696.3 (L†Ge$^+$, 5), 623.4 (L$^{†+}$, 40), 580.3 (L$^{†+}$-Pr^i, 75), 167.0 (Ph$_2$C$^+$, 100); anal. calcd. for $C_{49}H_{61}$GeNSi: C, 76.96 %; H, 8.04 %; N, 1.84 %; found: C, 76.81 %; H, 8.13 %; N, 1.91 %.

Preparation of [L†Ge(C_6H_{11})] 9. Using **1** (0.20 g, 0.14 mmol), and cyclohexene (0.36 mmol, 32 μL). The yellow product was recrystallised from 4 mL hexane, with additional cyclohexene (80 μL) added, at 4 ºC (95 mg, 42 %).

Alternative synthesis. To a suspension of flame-dried fine Mg turnings (22 mg, 0.89 mmol) in THF (15 mL) was added CyCl (89 μL, 0.75 mmol). The reaction mixture was then heated at 40 ºC for 4 h. The resultant suspension was cooled and filtered directly into a stirred solution of [L†GeCl] (0.50 g, 0.69 mmol) in THF (25 mL) at -80 ºC. The mixture was then warmed to ambient temperature, and stirred for a further 30 min, over which time the solution became bright yellow. An ^{1}H NMR spectroscopic analysis showed essentially quantitative conversion to [L†Ge(C_6H_{11})] by this time. All volatiles were subsequently removed *in vacuo*, and the residue extracted into hexane, then the extract filtered. Concentration of the filtrate to 2 mL *in vacuo*, and storage overnight at 4 ºC, resulted in the precipitation of **9** as a yellow-orange crystalline solid (390 mg, 73 %). M.p. 127-133 °C (melt); ^{1}H NMR (C_6D_6, 400 MHz, 298 K): δ = 0.82 (m, 1H, GeCH), 1.02 (d, $^3J_{HH}$ = 6.8 Hz, 6H, Ar†-*p*-CH(CH_3)$_2$), 1.10 (m, 4H, Ge(C_6H_{11})-CH_2), 1.24 (m, 6H, Ge(C_6H_{11})-CH_2), 1.29 (d, $^3J_{HH}$ = 7.6 Hz, 18H, SiCH(CH_3)$_2$), 1.71 (sept, $^3J_{HH}$ = 7.6 Hz, 3H, SiCH(CH$_3$)$_2$), 2.58 (sept, $^3J_{HH}$ = 6.8 Hz, 1H, Ar†-*p*-CH(CH$_3$)$_2$), 6.25 (s, 2H, CHPh$_2$), 6.93-7.36 (m, 22H, Ar-H); ^{13}C{^{1}H} NMR (C_6D_6, 75.5 MHz, 298 K): δ = 15.7 (SiCH(CH$_3$)$_2$), 20.0 (SiCH(CH$_3$)$_2$), 23.1 (Ar†-*p*-CH(CH$_3$)$_2$), 26.3 (Ge(C_6H_{11})-CH_2), 27.2 (Ge(C_6H_{11})-CH_2), 28.2 (Ge(C_6H_{11})-CH_2), 33.8 (Ar†-*p*-CH(CH$_3$)$_2$), 52.4 (CHPh$_2$), 52.8 (GeCH), 126.5, 127.1, 128.5, 129.0, 130.1, 130.8, 140.8, 143.5, 144.5, 145.0, 145.5, 148.1 (Ar-C); ^{29}Si{^{1}H} NMR (C_6D_6, 80 MHz, 298 K): δ = 7.8; IR, ν/cm^{-1} (ATR): 1600 (w), 1379 (m), 1225 (m), 1199 (m), 1031 (m), 876 (s), 834 (s), 734 (s), 729 (s), 656 (s); MS/EI *m/z* (%): 696.4 (L†Ge$^+$, 3), 623.4 (L$^{†+}$, 32), 580.3 (L$^{†+}$-Pr^i, 75), 167.0 (Ph$_2$C$^+$, 100); anal. calcd. for $C_{50}H_{63}$GeNSi: C, 77.11 %; H, 8.15 %; N, 1.80 %; found: C, 76.99 %; H, 8.26 %; N, 1.87 %.

Preparation of [L†Ge(C_8H_{15})] 10. Using **1** (0.20 g, 0.14 mmol), and cyclooctene (0.36 mmol, 46 μL). The yellow product was recrystallised from 4 mL hexane, in the presence of additional cyclooctene (120 μL), at -30 ºC (120 mg, 53 %). M.p. 112-118 °C (dec.); ^{1}H NMR (C_6D_6, 400 MHz, 298 K): δ = 1.00 (d, $^3J_{HH}$ = 6.8 Hz, 6H, Ar†-*p*-CH(CH_3)$_2$), 1.10-1.62 (br m, 15H, Ge(C_8H_{15})-CH_2/CH), 1.29 (br m, 18H, SiCH(CH_3)$_2$), 1.78 (sept, $^3J_{HH}$ = 7.6 Hz, 3H, SiCH(CH$_3$)$_2$), 2.56 (sept,

$^3J_{HH}$ = 6.8 Hz, 1H, Ar†-*p*-C*H*(CH$_3$)$_2$), 6.29 (s, 2H, C*H*Ph$_2$), 6.95-7.36 (m, 22H, Ar-*H*); $^{13}C\{^1H\}$ NMR (C$_6$D$_6$, 75.5 MHz, 298 K): δ = 15.8 (SiCH(*C*H$_3$)$_2$), 19.0 (Ge(C$_8$H$_{15}$)-C*H*$_2$), 19.9 (Si*C*H(CH$_3$)$_2$), 24.1 (Ar†-*p*-CH(*C*H$_3$)$_2$), 26.3, 26.9, 27.4 (Ge(C$_8$H$_{15}$)-C*H*$_2$), 33.8 (Ar†-*p*-*C*H(CH$_3$)$_2$), 52.0 (*C*HPh$_2$), 52.8 (Ge*C*H), 126.5, 127.1, 128.5, 128.8, 130.1, 130.2, 130.4, 130.8, 131.2, 145.0, 146.0, 147.6 (Ar-C); $^{29}Si\{^1H\}$ NMR (C$_6$D$_6$, 80 MHz, 298 K): δ = 7.2; IR, ν/cm^{-1} (ATR): 1599 (w), 1377 (m), 1224 (w), 1196 (m), 1115 (m), 1033 (m), 879 (s), 833 (s), 727 (s), 699 (s), 659 (s); MS/EI *m/z* (%): 696.3 (L†Ge$^+$, 18), 623.3 (L$^{\dagger+}$, 48), 580.3 (L$^{\dagger+}$-Pr^i, 100); anal. calcd. for C$_{52}$H$_{67}$GeNSi: C, 77.41 %; H, 8.37 %; N, 1.74 %; found: C, 77.34 %; H, 8.48 %; N, 1.81 %.

Preparation of [L†SnEt] 11.

Route (a): Using **2** (0.20 g, 0.13 mmol) and ethylene (1 atm.). The product was recrystallised at 4 ºC from a concentrated (~5 mL) hexane solution, yielding large yellow-orange blocks (125 mg, 60 %).

Route (b): Using [L†SnOBu^t] (0.30 g, 0.37 mmol), HBpin (60 µL, 0.41 mmol), and ethylene (1 atm.). The product was isolated as an analytically pure deep yellow powder, through removal of volatiles *in vacuo* from the hexane extract of the reaction mixture, and washing the solid residue with pentane (~10 mL), before drying *in vacuo* (210 mg, 73 %). M.p. 132-143 °C (melt); ^1H NMR (C$_6$D$_6$, 400 MHz, 298 K): δ = -0.82 (br, 2 H, SnC*H*$_2$), 0.83 (t, $^3J_{HH}$ = 7.2 Hz, 3H, SnCH$_2$C*H*$_3$), 1.04 (d, $^3J_{HH}$ = 6.8 Hz, 6H, Ar†-*p*-CH(C*H*$_3$)$_2$), 1.31 (d, $^3J_{HH}$ = 7.6 Hz, 18H, SiCH(C*H*$_3$)$_2$), 1.73 (sept, $^3J_{HH}$ = 7.6 Hz, 3H, SiC*H*(CH$_3$)$_2$), 2.61 (sept, $^3J_{HH}$ = 6.8 Hz, 1H, Ar†-*p*-C*H*(CH$_3$)$_2$), 6.37 (s, 2H, C*H*Ph$_2$), 6.91-7.32 (m, 22H, Ar-*H*); $^{13}C\{^1H\}$ NMR (C$_6$D$_6$, 75.5 MHz, 298 K): δ = 7.7 (SnCH$_2$*C*H$_3$), 14.9 (Si*C*H(CH$_3$)$_2$), 19.1 (SiCH(*C*H$_3$)$_2$), 24.0 (Ar†-*p*-CH(*C*H$_3$)$_2$), 33.7 (Ar†-*p*-*C*H(CH$_3$)$_2$), 49.1 (Sn*C*H$_2$CH$_3$), 51.9 (*C*HPh$_2$), 126.6, 127.6, 128.6, 129.7, 129.8, 129.9, 130.1, 140.8, 143.3, 145.0, 145.9, 150.9 (Ar-*C*); $^{29}Si\{^1H\}$ NMR (C$_6$D$_6$, 80 MHz, 298 K): δ = 6.2; IR, ν/cm^{-1} (ATR): 1597 (m), 1379 (m), 1221 (s), 1201 (s), 1116 (s), 1031 (m), 982 (m), 881 (s), 831 (s), 759 (s); MS/EI *m/z* (%): 770.5 (M$^+$, 1), 742.6 (L†Sn$^+$, 2), 167.2 (Ph$_2$C$^+$, 100); anal. calcd. for C$_{46}$H$_{57}$NSiSn: C, 71.68 %; H, 7.45 %; N, 1.82 %; found: C, 71.59 %; H, 7.51 %; N, 1.82 %.

Preparation of [L†Sn(C$_2$H$_4$Bu^t)] 12.

Route (a): Using **2** (0.20 mg, 0.13 mmol) and 3,3-dimethyl-1-butene (52 µL, 0.41 mmol). The product was recrystallised at 4 ºC from a concentrated (~5 mL) hexane solution, yielding large red-orange blocks (150 mg, 67 %).

Route (b): Using [L†SnOBu^t] (0.30 g, 0.37 mmol), HBpin (60 µL, 0.41 mmol), and 3,3-dimethyl-1-butene (71 µL, 0.56 mmol). The product was recrystallised at 4 ºC from a concentrated (~8 mL) hexane solution, yielding large red-orange blocks (200 mg, 66 %). M.p. 82-92 °C (melt); ^1H NMR

(C_6D_6, 400 MHz, 298 K): δ = -0.42 (br, 2H, SnC*H*$_2$), 0.67 (s, 9H, C(C*H*$_3$)$_3$), 0.98 (m, 2H, SnCH$_2$C*H*$_2$), 1.07 (d, $^3J_{HH}$ = 6.8 Hz, 6H, Ar†-*p*-CH(C*H*$_3$)$_2$), 1.31 (d, $^3J_{HH}$ = 7.6 Hz, 18H, SiCH(C*H*$_3$)$_2$), 1.67 (sept, $^3J_{HH}$ = 7.6 Hz, 3H, SiC*H*(CH$_3$)$_2$), 2.64 (sept, $^3J_{HH}$ = 6.8 Hz, 1H, Ar†-*p*-C*H*(CH$_3$)$_2$), 6.41 (s, 2H, C*H*Ph$_2$), 6.93-7.36 (m, 22H, Ar-*H*); $^{13}C\{^1H\}$ NMR (C_6D_6, 75.5 MHz, 298 K): δ = 15.9 (Si*C*H(CH$_3$)$_2$), 20.3 (SiCH(*C*H$_3$)$_2$), 24.4 (Ar†-*p*-CH(*C*H$_3$)$_2$), 29.3 (C(*C*H$_3$)$_3$), 32.7 (Ar†-*p*-*C*H(CH$_3$)$_2$), 33.7 (SnCH$_2$*C*H$_2$), 37.6 (Sn*C*H$_2$) , 51.6 (*C*HPh$_2$), 53.7 (*C*(CH$_3$)$_3$), 126.3, 127.6, 128.6, 129.7, 129.9, 130.1, 130.3, 139.7, 141.8, 145.9, 146.2, 151.0 (Ar-*C*); $^{29}Si\{^1H\}$ NMR (C_6D_6, 80 MHz, 298 K): δ = 5.9; IR, ν/cm^{-1} (ATR): 1598 (w), 1380 (m), 1363 (m), 1222 (m), 1155 (m), 1116 (m), 1031 (m), 878 (s), 830 (s), 759 (s); MS/EI *m/z* (%): 623.4 (L$^{†+}$, 55), 580.4 (L$^{†+}$-Pr^i, 100); anal. calcd. for $C_{50}H_{65}NSiSn$: C, 72.63 %; H, 7.92 %; N, 1.69 %; found: C, 72.49 %; H, 8.10 %; N, 1.79 %.

Preparation of [L†Sn(C$_5$H$_9$)] 13.

Route (a): Using **2** (0.25 g, 0.17 mmol) and cyclopentene (180 µL, 2.04 mmol). The product was recrystallised at -30 ºC from a concentrated (~6 mL) hexane solution, in the presence of additional cyclopenetene (150 µL), yielding large red-orange blocks (125 mg, 45 %).

Route (b): Using [L†SnOBu^t] (0.3 g, 0.37 mmol), HBpin (60 µL, 0.41 mmol), and cyclopentene (196 µL, 2.22 mmol). The product was recrystallised from a concentrated hexane solution (~5 mL), with additional cyclopentene (150 µL) added, at -30 ºC, yielding red-orange blocks (230 mg, 77 %). M.p. 96-106 ºC (dec.); ^1H NMR (C_6D_6, 400 MHz, 298 K): 1.03 (d, $^3J_{HH}$ = 6.8 Hz, 6H, Ar†-*p*-CH(C*H*$_3$)$_2$), 1.32 (m, 4H, Sn(C$_5$H$_9$)-C*H*$_2$), 1.36 (d, $^3J_{HH}$ = 7.0 Hz, 18H, SiCH(C*H*$_3$)$_2$), 1.51 (br m, 1H, SnC*H*), 1.57 (br m, 4H, Sn(C$_5$H$_9$)-C*H*$_2$), 1.83 (sept, $^3J_{HH}$ = 7.0 Hz, 3H, SiC*H*(CH$_3$)$_2$) 2.58 (sept, $^3J_{HH}$ = 6.8 Hz, 1H, Ar†-*p*-C*H*(CH$_3$)$_2$), 6.44 (s, 2H, Ph$_2$C*H*), 6.88-7.39 (m, 22H, Ar-*H*); $^{13}C\{^1H\}$ NMR (C_6D_6, 75.5 MHz, 298 K): δ = 16.5 (Si*C*H(CH$_3$)$_2$), 19.3 (SiCH(*C*H$_3$)$_2$), 23.2 (Sn*C*H), 24.6 (Ar†-*p*-CH(*C*H$_3$)$_2$), 26.9, 27.9 (SnCH(*C*$_2$H$_4$)$_2$), 33.8 (Ar†-*p*-*C*H(CH$_3$)$_2$), 52.3 (*C*HPh$_2$), 126.5, 126.6, 128.6, 129.6, 130.0, 130.1, 140.8, 142.5, 144.6, 145.0, 145.4, 149.6 (Ar-*C*); $^{29}Si\{^1H\}$ NMR (C_6D_6, 80 MHz, 298 K): δ = 4.7; IR, ν/cm^{-1} (ATR): 1598 (m), 1378 (m), 1222 (m), 1195 (m), 1113 (m), 1031 (m), 916 (m), 877 (s), 843 (s), 805 (s), 743 (s), 671 (s); MS/EI *m/z* (%): 810.7 (M$^+$, 1), 742.6 (L†Sn$^+$, 7), 167.2 (Ph$_2$C$^+$ 100); anal. calcd. for $C_{49}H_{61}NSiSn$: C, 72.58 %; H, 7.58 %; N, 1.73 %; found: C, 72.57 %; H, 7.67 %; N, 1.85 %.

Preparation of [L†Ge{C(Ph)=C(H)(Me)}] 14. Using **1** (0.25 g, 0.18 mmol), and 1-phenyl-1-propyne (0.43 mmol, 54 µL). The yellow compound was crystallised from 5 mL hexane at -30 ºC (120 mg, 44 %). M.p. 146-158 °C (dec.); ^1H NMR (C_6D_6, 400 MHz, 298 K): δ = 1.04 (d, $^3J_{HH}$ = 6.8 Hz, 6H, Ar†-*p*-CH(C*H*$_3$)$_2$), 1.18 (d, $^3J_{HH}$ = 7.6 Hz, 18H, SiCH(C*H*$_3$)$_2$), 1.66 (sept, $^3J_{HH}$ = 7.6 Hz,

3H, Si*CH*(CH$_3$)$_2$), 1.66 (br, 3H, Ge(Ph)C=C(H)C*H*$_3$), 2.61 (sept, $^3J_{HH}$ = 6.8 Hz, 1H, Ar†-*p*-C*H*(CH$_3$)$_2$), 4.18 (v br, 1H, Ge(Ph)C=C(*H*)Me), 6.32 (s, 2H, C*H*Ph$_2$), 6.84-7.47 (m, 27H, Ar-*H*); ^{13}C{^{1}H} NMR (C$_6$D$_6$, 75.5 MHz, 298 K): δ = 14.5 (Si*C*H(CH$_3$)$_2$), 19.7 (SiCH(*C*H$_3$)$_2$), 23.1 (GeC(Ph)=C(H)*C*H$_3$), 24.2 (Ar†-*p*-CH(*C*H$_3$)$_2$), 33.8 (Ar†-*p*-*C*H(CH$_3$)$_2$), 51.3 (*C*HPh$_2$), 80.5 (Ge*C*(Ph)=C(H)Me), 86.1 (GeC(Ph)=*C*(H)Me), 125.6, 126.4, 127.0, 128.5, 129.0, 129.9, 130.1, 130.8, 137.9, 138.1, 142.7, 143.4, 145.2, 145.8, 145.9, 147.9 (Ar-*C*); ^{29}Si{^{1}H} NMR (C$_6$D$_6$, 80 MHz, 298 K): δ = 10.7; IR, ν/cm^{-1} (ATR): 1597(m), 1364 (m), 1224 (w), 1195 (w), 1113 (m), 1074 (m), 1030 (m), 878 (s), 831 (s), 756 (s), 662 (s); MS/EI *m/z* (%): 623.7 (L†$^+$, 30), 167.2 (Ph$_2$C$^+$, 100). A reproducible microanalysis could not be obtained, as the compound consistently recrystallized with small amounts (*ca.* 5%) of the secondary amine, L†H, which could not be completely removed, despite several recrystallizations of the product.

Preparation of [L†Sn{C(Ph)=C(H)(Me)}] 15. This compound was synthesised *via* Route (a) only, using **2** (0.20 g, 0.13 mmol), and 1-phenyl-1-propyne (38 μL, 0.30 mmol). The yellow-orange product was recrystallised from hexane (~3 mL) at -30 ºC (105 mg, 47 %). M.p. 115-120 °C (dec.); ^{1}H NMR (C$_6$D$_6$, 400 MHz, 298 K): δ = 1.02 (d, $^3J_{HH}$ = 6.8 Hz, 6H, Ar†-*p*-CH(C*H*$_3$)$_2$), 1.21 (d, $^3J_{HH}$ = 7.6 Hz, 18H, SiCH(C*H*$_3$)$_2$), 1.56 (br d, $^3J_{HH}$ ~ 6 Hz, 3H, SnC(Ph)=C(H)C*H*$_3$), 1.77 (sept, $^3J_{HH}$ = 7.6 Hz, 3H, SiC*H*(CH$_3$)$_2$), 2.57 (sept, $^3J_{HH}$ = 6.8 Hz, 1H, Ar†-*p*-C*H*(CH$_3$)$_2$), 5.26 (br quart, $^3J_{HH}$ ~ 6 Hz, 1H, Sn-C(Ph)=C(*H*)Me), 6.39 (s, 2H, C*H*Ph$_2$), 6.90-7.35 (m, 27H, Ar-*H*); ^{13}C{^{1}H} NMR (C$_6$D$_6$, 75.5 MHz, 298 K): δ = 15.3 (Si*C*H(CH$_3$)$_2$), 16.6 (SnC(Ph)=C(H)*C*H$_3$), 19.5 (SiCH(*C*H$_3$)$_2$), 24.3 (Ar†-*p*-CH(*C*H$_3$)$_2$), 33.8 (Ar†-*p*-*C*H(CH$_3$)$_2$), 52.8 (*C*HPh$_2$), 82.2 (br, Sn*C*(Ph)=*C*(H)Me), 125.0, 126.6, 127.6, 128.6, 128.7, 129.7, 129.9, 130.1, 131.4, 131.5, 140.8, 142.5, 144.9, 145.0, 145.6, 148.6 (Ar-*C*); ^{29}Si{^{1}H} NMR (C$_6$D$_6$, 80 MHz, 298 K): δ = 6.8; IR, ν/cm^{-1} (ATR): 1597 (m), 1380 (m), 1257 (m), 1155 (m), 1071 (m), 1013 (m), 882 (s), 759 (s); MS/EI *m/z* (%): 742.6 (L†Sn$^+$, 3), 167.2 (Ph$_2$C$^+$, 100); anal. calcd. for C$_{53}$H$_{61}$NSiSn: C, 74.12 %; H, 7.16 %; N, 1.63 %; found: C, 73.99 %; H, 7.04 %; N, 1.69 %.

Preparation of [L†Ge(C$_8$H$_{13}$)] 16. Using **1** (0.20 g, 0.14 mmol), and 1,5-cyclooctadiene (0.36 mmol, 46 μL). The yellow product was recrystallised from 3 mL hexane, with additional 1,5-cyclooctadiene (120 μL) added, at -30 ºC (80 mg, 36 %). M.p. 108-112 °C (melt); ^{1}H NMR (C$_6$D$_6$, 400 MHz, 298 K): δ = 0.99 (d, $^3J_{HH}$ = 6.8 Hz, 6H, Ar†-*p*-CH(C*H*$_3$)$_2$), 1.29 (d, $^3J_{HH}$ = 7.6 Hz, 18H, SiCH(C*H*$_3$)$_2$), 1.40 (m, 1H, GeC*H*), 1.52-1.68 (br m, 10H, Ge(C$_8$H$_{13}$)-C*H*$_2$), 1.79 (sept, $^3J_{HH}$ = 7.6 Hz, 3H, SiC*H*(CH$_3$)$_2$), 2.56 (sept, $^3J_{HH}$ = 6.8 Hz, 1H, Ar†-*p*-C*H*(CH$_3$)$_2$), 4.23 (m, 1H, CH=C*H*), 4.99 (m, 1H, CH=C*H*), 6.29 (s, 2H, C*H*Ph$_2$), 6.96-7.37 (m, 22H, Ar-*H*); ^{13}C{^{1}H} NMR (C$_6$D$_6$, 75.5 MHz, 298 K): δ = 15.7 (Si*C*H(CH$_3$)$_2$), 19.9 (SiCH(*C*H$_3$)$_2$), 23.8 (Ge(C$_8$H$_{13}$)-*C*H$_2$), 24.1 (Ar†-*p*-

CH(CH_3)$_2$), 27.5 (Ge(C_8H_{13})-CH_2), 28.5 (Ge(C_8H_{13})-CH_2), 29.0 (Ge(C_8H_{13})-CH_2), 33.8 (Ar†-*p*-CH(CH_3)$_2$), 52.1 (CHPh$_2$), 53.3 (GeCH), 91.4 (br, 2xCH=CH), 126.5, 127.1, 128.5, 128.8, 129.0, 130.2, 131.1, 141.8, 143.6, 144.3, 145.8, 147.2 (Ar-C); ^{29}Si{^{1}H} NMR (C_6D_6, 80 MHz, 298 K): δ = 7.3; IR, ν/cm^{-1} (ATR): 1599 (m), 1380 (m), 1227 (m), 1118 (s), 879 (s), 793 (s), 718 (s), 661 (s); MS/EI *m/z* (%): 696.3 (M^+-Pr^i, 1), 623.3 (L$^{†+}$, 15), 580.4 (L$^{†+}$-Pr^i, 35) 167.2 (Ph_2C^+, 42). A reproducible microanalysis could not be obtained, as the compound consistently recrystallized with small amounts (*ca.* 5%) of secondary amine, L†H, which could not be completely removed, despite several recrystallizations of the product.

Preparation of [L†Ge{C_2H_4C(H)Me$_2$}] 17. Using **1** (0.20 g, 0.14 mmol) and 2-methyl-2-butene (0.36 mmol, 38 μL). The yellow product was recrystallised from a concentrated hexane solution (3 mL) at 4 ºC (140 mg, 65 %). M.p. 142-150 °C (melt); ^{1}H NMR (C_6D_6, 400 MHz, 298 K): δ = -0.89 (br, 2H, GeCH_2), 0.58 (d, $^3J_{HH}$ = 6.0 Hz, 6H, Ge(CH_2)$_2$CH(CH_3)$_2$), 0.63 (m, 1H, Ge(CH_2)$_2$CH(CH_3)$_2$), 0.81 (br m, 2H, GeCH$_2$CH_2), 1.04 (d, $^3J_{HH}$ = 6.8 Hz, 6H, Ar†-*p*-CH(CH_3)$_2$), 1.26 (d, $^3J_{HH}$ = 7.6 Hz, 18H, SiCH(CH_3)$_2$), 1.73 (sept, $^3J_{HH}$ = 7.6 Hz, 3H, SiCH(CH_3)$_2$), 2.60 (sept, $^3J_{HH}$ = 6.8 Hz, 1H, Ar†-*p*-CH(CH_3)$_2$), 6.17 (s, 2H, CHPh$_2$), 7.95-7.44 (m, 22H, Ar-H); ^{13}C{^{1}H} NMR (C_6D_6, 75.5 MHz, 298 K): δ = 8.0 (GeCH$_2$$CH_3$), 14.3 (Si$C$H($CH_3$)$_2$), 19.7 (SiCH($CH_3$)$_2$), 22.7 (Ge($CH_2$)$_2$CH($CH_3$)$_2$), 24.1 (Ar†-*p*-CH($CH_3$)$_2$), 31.1 (GeCH$_2$$CH_2$), 32.7 (Ge($CH_2$)$_2$$C$H($CH_3$)$_2$), 33.7 (Ar†-*p*-$C$H($CH_3$)$_2$), 43.9 (Ge$CH_2$), 51.2 ($CHPh_2$), 126.3, 127.1, 128.2, 128.9, 129.5, 129.7, 129.9, 137.7, 142.7, 145.6, 146.2, 149.0 (Ar-C); ^{29}Si{^{1}H} NMR (C_6D_6, 80 MHz, 298 K): δ = 11.1; IR, ν/cm^{-1} (ATR): 1598 (m), 1380 (m), 1362 (m), 1258 (m), 1225 (m), 1114 (m), 1073 (m), 1032 (m), 877 (s), 811 (s), 759 (s), 727 (s), 658 (s); MS/EI *m/z* (%): 695.6 (L†Ge$^+$, 4), 580.6 (L$^{†+}$-Pr^i, 23), 167.2 (PhC$^+$, 100); anal. calcd. for $C_{49}H_{63}$GeNSi: C, 76.76 %; H, 8.28 %; N, 1.83 %; found: C, 76.61 %; H, 8.37 %; N, 2.01 %.

Preparation of [L†Ge(C_5H_9)(H)Cl] 20. The reaction of 1 equivalent of HCl in diethyl ether (1M solution) with an ethereal solution of compound **8** at -80 °C, followed by warming to ambient temperature and work-up, led to the isolation of a few colourless crystals of the title compound. Given the very low yield of the compound (< 2%), no spectroscopic or other data could be obtained for the compound. However, its X-ray crystal structure unambiguously identifies the molecular connectivity of the compound (see main text).

Preparation of [L†Ge(Et)(H)NH_2] 21. A yellow solution of [L†GeEt] **5** (200 mg, 0.28 mmol) in toluene was cooled to -25 ºC in a Schlenk flask, and dry NH_3 gas (8.4 mL, ~0.35 mmol) carefully introduced above the solution with a gas tight syringe, under a slow flow of N_2. The flask was

sealed, and the reaction mixture rapidly stirred. The yellow colour of the solution quickly dissipated to colourless. The reaction mixture was then warmed to ambient temperature, stirred for 1 h, and all volatiles subsequently removed *in vacuo*. The solid residue was extracted in 10 mL of hexane, the extract filtered, and the filtrate concentrated *in vacuo* to ~3 mL. Storage of the solution at -30 ºC for 24 h resulted in the formation of large colourless crystals of **21** (125 mg, 60 %). M.p. 160-166 °C (melt); ^{1}H NMR (C_6D_6, 400 MHz, 298 K): δ = -0.04 (br d, $^{3}J_{HH}$ ~ 4 Hz, 2H, N*H*$_2$), 0.20 (m, 1H, GeC*H*$_2$), 0.37 (m, 1H, GeC*H*$_2$), 0.55 (virt. t, $^{3}J_{HH}$ = 7.2 Hz, 3H, GeCH$_2$C*H*$_3$), 1.01 (d, $^{3}J_{HH}$ = 6.8 Hz, 6H, Ar†-*p*-CH(C*H*$_3$)$_2$), 1.21 (d, $^{3}J_{HH}$ = 7.6 Hz, 9H, SiCH(C*H*$_3$)$_2$), 1.25 (d, $^{3}J_{HH}$ = 7.6 Hz, 9H, SiCH(C*H*$_3$)$_2$), 1.50 (sept, $^{3}J_{HH}$ = 7.6 Hz, 3H, SiC*H*(CH$_3$)$_2$), 2.55 (sept, $^{3}J_{HH}$ = 6.8 Hz, 1H, Ar†-*p*-C*H*(CH$_3$)$_2$), 5.52 (m, 1H, Ge*H*), 6.50 (s, 1H, C*H*Ph$_2$), 6.51 (s, 1H, C*H*Ph$_2$), 6.97-7.46 (m, 22H, Ar-*H*); ^{13}C{^{1}H} NMR (C_6D_6, 75.5 MHz, 298 K): δ = 8.6 (GeCH$_2$*C*H$_3$), 14.6 (Ar†-*p*-CH(*C*H$_3$)$_2$), 19.1 (SiCH(*C*H$_3$)$_2$), 19.7 (SiCH(*C*H$_3$)$_2$), 23.9 (br, Si*C*H(CH$_3$)$_2$), 33.6 (Ar†-*p*-*C*H(CH$_3$)$_2$), 51.2 (*C*HPh$_2$),, 51.3 (*C*HPh$_2$), 52.8 (Ge*C*H$_2$CH$_3$), 126.3, 126.6, 126.8, 127.6, 128.6, 128.7, 129.9, 130.0, 130.1, 130.8, 130.9, 140.8, 141.4, 142.5, 142.6, 143.1, 143.3. 144.9, 145.1, 146.3, 146.5, 146.8 (Ar-*C*); ^{29}Si{^{1}H} NMR (C_6D_6, 80 MHz, 298 K): δ = 7.6; IR, ν/cm^{-1} (ATR): 3440, 3360 (w, NH$_2$), 2079 (m, Ge-H), 1598 (m), 1379 (m), 1364 (m), 1118 (m), 1032 (m), 909 (s), 880 (s), 853 (m), 744 (s), 725 (s); MS/EI *m/z* (%): 712.7 (M^{+},1), 696.6 (L†Ge^{+}, 8), 167.2 (Ph$_2$C^{+}, 100); anal. calcd. for $C_{46}H_{60}GeN_2Si$: C, 74.49 %; H, 8.15 %; N, 3.78 %; found: C, 74.59 %; H, 8.06 %; N, 3.62 %.

Preparation of [L†Ge(Et)(H)OEt] 22. To a yellow solution of [L†GeEt] (0.25 g, 0.34 mmol) in toluene (20 mL) at -80 ºC was added EtOH (20 μL, 37 mmol) using a micro pipettor. The reaction mixture was then warmed to ambient temperature with stirring, over which time the mixture became colourless. All volatiles were subsequently removed *in vacuo* and the residue extracted into diethyl ether (7 mL), and the extract filtered. Concentration of the filtrate to 2 mL and storage at 4 ºC for 2 days resulted in the formation of large colourless blocks of **22** (180 mg, 69 %). M.p. 143-152 °C (melt); ^{1}H NMR (C_6D_6, 400 MHz, 298 K): δ = -0.60 (m, 1H, GeC*H*$_2$), -0.14 (m, 1H, GeC*H*$_2$), 0.33 (virt. t, $^{3}J_{HH}$ = 7.2 Hz, 3H, GeCH$_2$C*H*$_3$), 1.05 (d, $^{3}J_{HH}$ = 6.8 Hz, 6H, Ar†-*p*-CH(C*H*$_3$)$_2$), 1.15 (d, $^{3}J_{HH}$ = 7.6 Hz, 9H, SiCH(C*H*$_3$)$_2$), 1.31 (d, $^{3}J_{HH}$ = 7.6 Hz, 9H, SiCH(C*H*$_3$)$_2$), 1.28 (virt t, 3H, $^{3}J_{HH}$ = 6.8 Hz, GeOCH$_2$C*H*$_3$), 1.44 (sept, $^{3}J_{HH}$ = 7.6 Hz, 3H, SiC*H*(CH$_3$)$_2$), 2.60 (sept, $^{3}J_{HH}$ = 6.8 Hz, 1H, Ar†-*p*-C*H*(CH$_3$)$_2$), 3.67 (m, 1H, GeOC*H*$_2$), 3.81 (m, 1H, GeOC*H*$_2$), 5.97 (m, 1H, Ge*H*), 6.54 (s, 2H, C*H*Ph$_2$), 6.95-7.77 (m, 22H, Ar-*H*); ^{13}C{^{1}H} NMR (C_6D_6, 75.5 MHz, 298 K): δ = 7.7 (GeCH$_2$*C*H$_3$), 9.9 (GeOCH$_2$*C*H$_3$), 13.9 (Si*C*H(CH$_3$)$_2$), 19.3 (SiCH(*C*H$_3$)$_2$), 19.6 (SiCH(*C*H$_3$)$_2$), 23.9 (Ar†-*p*-CH(*C*H$_3$)$_2$), 33.7 (Ar†-*p*-*C*H(CH$_3$)$_2$), 50.8 (Ge*C*H$_2$CH$_3$), 51.6 (*C*HPh$_2$), 61.9 (GeO*C*H$_2$CH$_3$), 126.2, 126.4, 126.5, 126.9, 128.0, 128.1, 128.6, 129.5, 129.7, 129.8, 129.9, 130.1, 130.8, 131.5, 142.2, 142.3, 143.6, 144.7, 145.0, 145.9, 146.2, 146.7 (Ar-*C*); ^{29}Si{^{1}H} NMR (C_6D_6, 80 MHz, 298

K): δ = 8.4; IR, ν/cm^{-1} (ATR): 2033 (m, Ge-H), 1599 (m), 1388 (m), 1365 (s), 1229 (s), 1158 (m), 1065 (m), 890 (m), 840 (m), 756 (s), 682(s), 659 (s); MS/EI *m/z* (%): 728.6 (M^+-Pr^i, 16), 580.6 (L$^{\dagger+}$-Pr^i, 41), 167.2 (Ph_2C^+, 100); anal. calcd. for $C_{48}H_{63}GeNOSi$: C, 74.80 %; H, 8.24 %; N, 1.82 %; found: C, 74.77 %; H, 8.08 %; N, 1.94 %.

Preparation of [L†Sn(OTEMP)] 23. A solution of TEMPOH (214 mg, 0.41 mmol) in toluene (10 mL) was added to a solution of [L†SnEt] (300 mg, 0.39 mmol) in toluene (20 mL) at -80 ºC. The reaction mixture was warmed to ambient temperature over 18 h, and all volatiles subsequently removed *in vacuo*. The residue was extracted into warm hexane (20 mL), and the extract filtered. Concentration of the filtrate to ~7 mL, and storage at -30 ºC, yielded **23** as colourless crystalline blocks (160 mg, 36 %). M.p. 115-120 °C (dec.); ^{1}H NMR (C_6D_6, 400 MHz, 298 K): δ = 0.77 (br, 6H, TEMPO-CH_3), 0.97 (d, $^3J_{HH}$ = 6.8 Hz, 6H, Ar†-*p*-CH(CH_3)$_2$), 1.12 (br, 6H, TEMPO-CH_3), 1.37 (br, 6H, TEMPO-CH_2), 1.60 (br, 18H, SiCH(CH_3)$_2$), 2.03 (sept, $^3J_{HH}$ = 7.6 Hz, 3H, SiCH(CH$_3$)$_2$), 2.52 (sept, $^3J_{HH}$ = 6.8 Hz, 1H, Ar†-*p*-CH(CH$_3$)$_2$), 6.56 (s, 2H, CHPh$_2$), 6.84 (m, 2H, Ar†-*m*-CH), 6.99-7.36 (m, 20H, Ar-H); ^{13}C{^{1}H} NMR (C_6D_6, 75.5 MHz, 298 K): δ = 14.7 (br, SiCH(CH$_3$)$_2$), 19.1 (TEMPO-CH$_2$), 19.7 (br, SiCH(CH$_3$)$_2$), 24.2 (Ar†-*p*-CH(CH$_3$)$_2$), 33.7 (Ar†-*p*-CH(CH$_3$)$_2$), 36.2 (br, TEMPO-CH$_3$), 40.2 (TEMPO-CH$_2$), 51.8 (CHPh$_2$), 58.4 (TEMPO-NC), 126.5, 127.6, 127.4, 128.7, 128.7, 129.7, 129.9, 130.1, 131.4, 131.5, 140.8, 142.5, 144.9, 145.0, 145.6, 148.6 (Ar-C); ^{29}Si{^{1}H} NMR (C_6D_6, 80 MHz, 298 K): δ = 26.1; ^{119}Sn{^{1}H} NMR (C_6D_6, 149 MHz, 298 K): δ = 177.3; IR, ν/cm^{-1} (ATR): 1598 (m), 1296 (m), 1221 (m), 1200 (m), 1117 (m), 1032 (m), 917 (m), 878 (s), 830 (s), 754 (m); MS/EI *m/z* (%): 623.6 (L$^{\dagger+}$, 20), 167.2 (Ph_2C^+, 100); anal. calcd. for $C_{53}H_{70}N_2OSiSn$: C, 70.89 %; H, 7.86 %; N, 3.12 %; found: C, 71.12 %; H, 7.77 %; N, 2.99 %.

Preparation of [L†SnOBu^t] 24. The reaction of [L†SnEt] in C_6D_6 with one equivalent of *tert*-butanol was carried out in a J. Young's NMR tube, and the course of the reaction monitored by ^{1}H NMR spectroscopy. This showed the complete consumption of [L†SnEt] and the formation of [L†SnOBu^t] and L†H in a ratio of approximately 70:30. A signal for generated ethane was also observed in the spectrum. The chemical shifts of the signals for [L†SnOBu^t] were at identical chemical shifts to those previously reported for this compound.[3]

Preparation of [{L†(C_6H_{11})Ge(μ-O)}$_2$] 25. Dry O_2 was introduced to the headspace (*ca.* 50 mL) of a Schlenk flask containing a yellow solution of [L†Ge(C_6H_{11})] **9** (0.15 g, 0.19 mmol) in diethyl ether (20 mL) at -80 ºC. The flask was then sealed, and the mixture stirred for 10 min, over which time the solution became colourless. The solution was warmed to ambient temperature, and subsequently concentrated *in vacuo* to 5 mL. Storage of the solution at 4 ºC for 18 h yielding

colourless crystalline blocks of **25** (50 mg, 33 %). M.p. 182-190 °C (melt); ^{1}H NMR (C_6D_6, 400 MHz, 298 K): δ = 0.19 (br m, 2H, Cy-C*H*$_2$), 1.17 (d, $^{3}J_{HH}$ = 7.6 Hz, 18H, SiCH(C*H*$_3$)$_2$), 1.20 (d, $^{3}J_{HH}$ = 6.8 Hz, 6H, Ar†-*p*-CH(C*H*$_3$)$_2$), 1.19-1.46 (br m, 8H, Cy-C*H*$_2$), 1.62 (sept, $^{3}J_{HH}$ = 7.6 Hz, 3H, SiC*H*(CH$_3$)$_2$), 1.87 (br m, 1H, Ge-C*H*), 2.80 (sept, $^{3}J_{HH}$ = 6.8 Hz, 1H, Ar†-*p*-C*H*(CH$_3$)$_2$), 6.70 (s, 2H, C*H*Ph$_2$), 6.98-7.93 (m, 22H, Ar-*H*); ^{13}C{^{1}H} NMR (C_6D_6, 75.5 MHz, 298 K): δ = 16.1 (Si*C*H(CH$_3$)$_2$), 21.0 (Cy-*C*H$_2$), 21.4 (SiCH(*C*H$_3$)$_2$), 24.8 (Ar†-*p*-CH(*C*H$_3$)$_2$), 25.6 (Cy-*C*H$_2$), 27.8 (Cy-*C*H$_2$), 28.7 (Cy-*C*H), 34.5 (Ar†-*p*-*C*H(CH$_3$)$_2$), 52.0 (*C*HPh$_2$), 126.8, 127.3, 128.5, 129.4, 130.5, 131.3, 132.0, 142.8, 144.3, 145.8, 146.6, 148.6 (Ar-C); ^{29}Si{^{1}H} NMR (C_6D_6, 80 MHz, 298 K): δ = 12.9; IR, ν/cm^{-1} (ATR): 1599 (w), 1381 (m), 1260 (m), 1156 (m), 1112 (m), 1074 (m), 1032 (m), 881 (s), 805 (s), 743 (s), 662 (s); MS/EI *m/z* (%): 623.7 (L$^{\dagger+}$, 20), 167.2 (Ph_2C^+, 100); anal. calcd. for $C_{100}H_{126}Ge_2N_2O_2Si_2$: C, 75.56 %; H, 7.99 %; N, 1.76 %; found: C, 75.47 %; H, 8.13 %; N, 1.86 %.

Preparation of [{L†(Bu^{t}C$_2$H$_4$)Sn(μ-O)}$_2$] 26. Dry O_2 was introduced to the headspace (*ca.* 50 mL) of a Schlenk flask containing a stired solution of [L†Sn(C$_2$H$_4$Bu^{t})] **12** (0.15 g, 0.18 mmol) in toluene (20 mL) at ambient temperature, and the flask subsequently sealed. The reaction solution rapidly became colourless, and after 10 min stirring, it was concentrated *in vacuo* to 5 mL, then stored at 4 ^{o}C for 18 h to give **26** as colourless crystalline blocks (75 mg, 49 %). M.p. 147-153 °C (melt); ^{1}H NMR (C_6D_6, 400 MHz, 298 K): δ = -0.42 (br, 2H, SnC*H*$_2$), 0.67 (s, 9H, C(C*H*$_3$)$_3$), 0.98 (m, 2H, SnCH$_2$C*H*$_2$), 1.07 (d, $^{3}J_{HH}$ = 6.8 Hz, 6H, Ar†-*p*-CH(C*H*$_3$)$_2$), 1.31 (d, $^{3}J_{HH}$ = 7.6 Hz, 18H, SiCH(C*H*$_3$)$_2$), 1.67 (sept, $^{3}J_{HH}$ = 7.6 Hz, 3H, SiC*H*(CH$_3$)$_2$), 2.64 (sept, $^{3}J_{HH}$ = 6.8 Hz, 1H, Ar†-*p*-C*H*(CH$_3$)$_2$), 6.41 (s, 2H, C*H*Ph$_2$), 6.93-7.36 (m, 22H, Ar-*H*); ^{13}C{^{1}H} NMR (C_6D_6, 75.5 MHz, 298 K): δ = 15.9 (Si*C*H(CH$_3$)$_2$), 20.3 (SiCH(*C*H$_3$)$_2$), 24.4 (Ar†-*p*-CH(*C*H$_3$)$_2$), 29.3 (C(*C*H$_3$)$_3$), 32.7 (Ar†-*p*-*C*H(CH$_3$)$_2$), 33.7 (SnCH$_2$*C*H$_2$), 37.6 (Sn*C*H$_2$), 51.6 (*C*HPh$_2$), 53.7 (*C*(CH$_3$)$_3$), 126.3, 127.6, 128.6, 129.7, 129.9, 130.1, 130.3, 139.7, 141.8, 145.9, 146.2, 151.0 (Ar-*C*); ^{29}Si{^{1}H} NMR (C_6D_6, 80 MHz, 298 K): δ = 5.9; IR, ν/cm^{-1} (ATR): 1598 (w), 1380 (m), 1363 (m), 1222 (m), 1155 (m), 1116 (m), 1031 (m), 878 (s), 830 (s), 759 (s); MS/EI *m/z* (%): 623.4 (L$^{\dagger+}$, 55), 580.4 (L$^{\dagger+}$-Pr^{i}, 100); anal. calcd. for $C_{100}H_{130}N_2O_2Si_2Sn_2\cdot(C_6H_{14})_2$: C, 72.41 %; H, 8.57 %; N, 1.51 %; found: C, 72.49 %; H, 8.10 %; N, 1.79 %.

Method for the van't Hoff analysis of the solution state equilibrium between 3, cyclohexene, and 9.

A sample of [L†Ge(C$_6$H$_{11}$)] **9** (20 mg), prepared through the addition of (C$_6$H$_{11}$)MgBr to [L†GeCl] (see above), was dissolved in C_6D_6 (0.5 mL), yielding a solution of concentration 51.4 mM. The solution was warmed to 304 K in a 600 MHz NMR machine fitted with a variable temperature

probe, and the solution held at this temperature for 10 minutes. Collecting 3 sequential ^{1}H NMR spectra over the course of 6 minutes confirmed that the composition of the C_6D_6 sample remained constant after this time, i.e. the equilibrium between **9**, **3** (and **1**), and cyclohexene had reached a steady state. Following this, the sample was warmed in 2 K increments, held at the relevant temperature for 10 minutes, and an ^{1}H NMR spectrum collected. This was carried out over the temperature range of 304 K–314 K, giving six ^{1}H NMR spectra. The relative concentration of **9**, **3** (and **1**), and cyclohexene was deduced through the relative integrations of the alkenic proton signal of cyclohexene, and the C*H*Ph$_2$ protons of **9** and **3** (and **1**). The actual concentration of each species was then determined from the initial solution concentration of **9** (51.4 mM). The values were then used to calculate the equilibrium constant (K$_{eq}$) at each temperature, according to the following equation:

$$K_{eq} = \frac{[L^{\dagger}GeCyHex]}{[L^{\dagger}GeH][cyclohexene]}$$

Calculating K$_{eq}$ for the temperature range 304 K–314 K allows for calculation of ΔH° and ΔS° values for the forward reaction, using the following equation:

$$lnK_{eq} = \frac{-\Delta H^{o}}{RT} + \frac{\Delta S^{o}}{R}$$

Plotting lnK$_{eq}$ against 1/T gives a straight line graph, the slope of which is equal to $\frac{-\Delta H^{o}}{R}$, with the *y* intercept being equal to $\frac{\Delta S^{o}}{R}$, from which values for ΔH° and ΔS° can be attained, respectively. Using these values, ΔG° can be calculated for any temperature using the equation:

$$\Delta G^{o} = \Delta H^{o} - T\Delta S^{o}$$

2. X-Ray Crystallography

Crystals of **5**, **6**, **8-10**, **11**, **13-17**, **20**, **22**, **23**, **25**, **26**, [L†Ge(μ-C_2H_4)GeL†] and [{L†(PhC_2H_4)Ge(μ-O)}$_2$] suitable for X-ray structural determination were mounted in silicone oil. Crystallographic measurements were made using either an Oxford Gemini Ultra diffractometer using a graphite monochromator with Mo Kα radiation (λ = 0.71073 Å) or Cu Kα radiation (1.54180 Å); or the MX1 beamline of the Australian Synchrotron (λ = 0.71080 Å or λ = 0.71090 Å). The software package Blu-Ice[5] was used for synchrotron data acquisition, while the program XDS[6] was employed for synchrotron data reduction. All structures were solved by direct methods and refined on F^2 by full matrix least squares (SHELX97[7]) using all unique data. Hydrogen atoms are included in calculated positions (riding model), except the hydride ligands of **20** and **22**, the atomic displacement parameters of which were refined isotropically. Two molecules of **23** and **26** were refined in the asymmetric units of their crystal structures. No significant geometric differences were found between them. Compound **9** was refined as a racemic twin, the absolute structure parameter of which converged to 0.393(9). Crystal data, details of data collections and refinements for all structures can be found in their CIF files and are summarized in Table S1.

Table S1. Crystal data for **5**, **6**, **8-10**, **11**, **13-17**, **20**, **22**, **23**, **25**, **26**, [L†Ge(μ-C_2H_4)GeL†] and [{L†(PhC_2H_4)Ge(μ-O)}$_2$].

	**5**	**6**	**8**	**9**	**10**
empirical formula	$C_{46}H_{57}GeNSi$	$C_{52}H_{61}GeNSi$	$C_{49}H_{61}GeNSi$	$C_{50}H_{63}GeNSi$	$C_{52}H_{67}GeNSi$
formula weight	724.61	800.70	764.67	778.69	806.75
crystal system	triclinic	monoclinic	monoclinic	monoclinic	monoclinic
space group	*P*-1	$P2_1/n$	$P2_1/c$	$P2_1$	$P2_1/n$
a (Å)	10.783(2)	11.5430(4)	10.319(2)	10.2741(5)	9.942(2)
b (Å)	11.465(2)	23.7164(9)	18.286(4)	14.6818(7)	15.413(3)
c (Å)	16.298(3)	16.3022(6)	22.437(5)	14.6417(7)	28.793(6)
α (º)	97.01(3)	90	90	90	90
β (º)	91.76(3)	97.9790(10)	91.27(3)	102.077(3)	97.18(3)
γ (º)	92.77(3)	90	90	90	90
V (Å^3)	1996.1(7)	4419.7(3)	4232.7(15)	2159.71(18)	4377.6(15)
Z	2	4	4	2	4
T (K)	100(2)	123(2)	100(2)	123(2)	100(2)
ρ_{calcd} (g·cm^3)	1.206	1.203	1.200	1.197	1.224
μ (mm^{-1})	0.829	0.755	0.785	0.771	0.763
F(000)	772	1704	1632	832	1728
reflns collected	25550	38192	22341	34220	69020
unique reflns	6617	12845	8201	8466	9523
R_{int}	0.0495	0.0570	0.1448	0.0585	0.0799
R1 [I > 2σ(I)]	0.0405	0.0477	0.0678	0.0426	0.0501
wR2 (all data)	0.0996	0.1088	0.1968	0.0965	0.1476
largest peak and hole (e·Å^{-3})	0.58, -0.78	0.51, -0.47	0.534, -2.079	0.59, -0.38	0.60, -0.79
CCDC no.	1422727	1422728	1422729	1422730	1422731

	**11**	**13**·(hexane)$_{0.5}$	**14**	**15**	**16**
empirical formula	$C_{46}H_{57}NSiSn$	$C_{52}H_{68}NSiSn$	$C_{53}H_{61}GeNSi$	$C_{53}H_{61}NSiSn$	$C_{52}H_{65}GeNSi$
formula weight	770.73	853.85	812.71	858.81	804.73
crystal system	triclinic	triclinic	monoclinic	monoclinic	monoclinic
space group	*P*-1	*P*-1	$P2_1/n$	$P2_1/n$	$P2_1/n$
a (Å)	10.673(2)	10.2591(9)	10.0813(17)	10.1088(10)	10.0030(6)
b (Å)	12.534(3)	13.6505(10)	15.796(3)	15.7379(15)	15.4017(12)
c (Å)	15.493(3)	17.3164(15)	28.096(4)	28.106(2)	28.920(2)
α (º)	82.27(3)	99.111(7)	90	90	90
β (º)	85.09(3)	105.293(8)	96.916(13)	96.853(8)	97.698(2)
γ (º)	73.63(3)	95.189(7)	90	90	90
V (Å^3)	1968.0(8)	2287.3(3)	4441.7(13)	4439.5(7)	4415.4(5)
Z	2	2	4	4	4
T (K)	100(2)	123(2)	123(2)	123(2)	100(2)
ρ_{calcd} (g·cm^3)	1.301	1.240	1.215	1.285	1.211
μ (mm^{-1})	0.712	0.619	0.753	0.639	0.756
F(000)	808	902	1728	1800	1720
reflns collected	22483	17097	21378	21635	38995
unique reflns	6158	8971	8251	8243	7515
R_{int}	0.062	0.0927	0.1932	0.0598	0.0512
R1 [I > 2σ(I)]	0.0396	0.0668	0.0925	0.0813	0.0452
wR2 (all data)	0.1052	0.1259	0.2723	0.1985	0.1155
largest peak and hole (e·Å^{-3})	0.81, -0.72	1.10, -1.01	1.02, -1.18	2.39, -0.85	0.69, -0.44
CCDC no.	1422732	1422733	1422734	1422735	1422736

	**17**	**20**	**22**	**23**	**25**·(hexane)
empirical formula	$C_{49}H_{63}GeNSi$	$C_{49}H_{62}ClGeNSi$	$C_{48}H_{63}GeNOSi$	$C_{53}H_{70}N_2OSiSn$	$C_{112}H_{154}Ge_2N_2O_2Si_2$
formula weight	766.68	801.13	770.67	897.89	1761.73
crystal system	monoclinic	triclinic	triclinic	triclinic	monoclinic
space group	$P2_1/n$	*P*-1	*P*-1	*P*-1	$P2_1/n$
a (Å)	11.3740(4)	10.731(2)	10.3990(4)	14.604(3)	15.0392(3)
b (Å)	23.3065(8)	14.446(3)	14.2109(5)	15.840(3)	12.4994(2)
c (Å)	16.3439(5)	15.647(3)	15.4524(5)	20.773(4)	26.2128(4)
α (º)	90	77.83(3)	90.4830(10)	88.61(3)	90
β (º)	97.891(2)	78.54(3)	101.0260(10)	82.84(3)	93.127(2)
γ (º)	90	69.06(3)	105.1180(10)	84.99(3)	90
V (Å^3)	4291.5(2)	2194.1(8)	2159.71(13)	4749.2(17)	4920.17(15)
Z	4	2	2	4	2
T (K)	123(2)	100(2)	123(2)	100(2)	123(2)
ρ_{calcd} (g·cm^3)	1.187	1.213	1.185	1.256	1.189
μ (mm^{-1})	0.775	0.819	0.772	0.602	1.359
F(000)	1640	852	824	1896	1896
reflns collected	88866	14376	33814	72300	37623
unique reflns	8398	7198	8425	18572	8723
R_{int}	0.0835	0.0826	0.0361	0.0450	0.0297
R1 [I > 2σ(I)]	0.0457	0.0635	0.0440	0.0340	0.0391
wR2 (all data)	0.1093	0.1805	0.1127	0.0877	0.1043
largest peak and hole (e·Å^{-3})	0.85, -0.43	1.18, -1.02	1.18, -0.34	0.95, -0.86	0.66, -0.58
CCDC no.	1422737	1422738	1422739	1422740	1422741

	**26**·(hexane)	$[L^{\dagger}Ge(\mu\text{-}C_2H_4)GeL^{\dagger}]\cdot(hexane)_{0.25}$	$[\{L^{\dagger}(PhC_2H_4)Ge(\mu\text{-}O)\}_2]$
empirical formula	$C_{112}H_{158}N_2O_2Si_2Sn_2$	$C_{91.5}H_{111.5}Ge_2N_2Si_2$	$C_{104}H_{122}Ge_2N_2O_2Si_2$
formula weight	1857.96	1440.69	1633.40
crystal system	triclinic	triclinic	monoclinic
space group	*P*-1	*P*-1	$P2_1/n$
a (Å)	15.4827(8)	10.806(2)	13.373(3)
b (Å)	16.4448(9)	18.897(4)	21.643(4)
c (Å)	23.7823(11)	21.557(4)	15.884(3)
α (º)	72.311(4)	108.61(3)	90
β (º)	88.259(4)	90.43(3)	102.62(3)
γ (º)	62.908(5)	103.93(3)	90
V ($Å^3$)	5094.1(5)	4032.0(14)	4486.3(16)
Z	2	2	2
T (K)	123(2)	100(2)	100(2)
ρ_{calcd} (g·cm^3)	1.211	1.187	1.209
μ (mm^{-1})	0.563	0.820	0.747
F(000)	1976	1533	1736
reflns collected	46935	52997	59686
unique reflns	18919	13668	8072
R_{int}	0.0474	0.0750	0.0947
R1 [I > 2σ(I)]	0.0495	0.0609	0.0699
wR2 (all data)	0.1342	0.1722	0.1796
largest peak and hole (e·$Å^{-3}$)	1.44, -0.88	1.19, -0.83	0.99, -0.74
CCDC no.	1422742	1422725	1422726

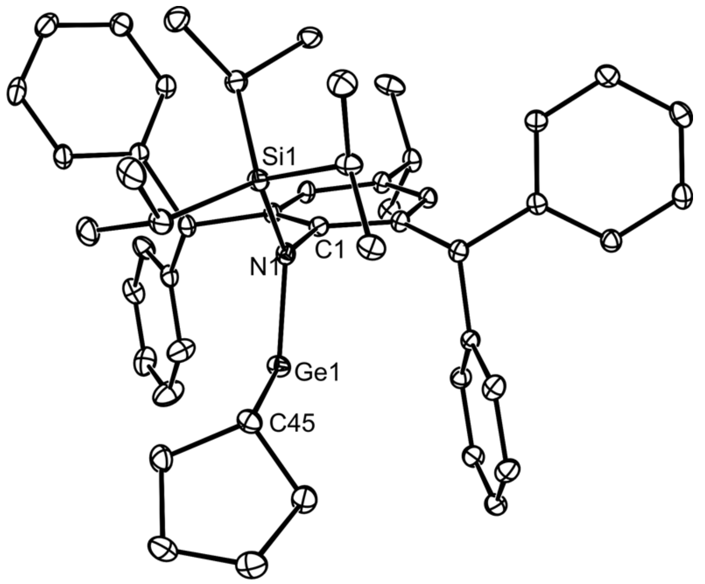

Figure S1. ORTEP diagram of compound **8** (25% thermal ellipsoids; hydrogen atoms omitted). Selected bond lengths (Å) and angles (º): Ge(1)-N(1) 1.885(3), Ge(1)-C(45) 1.999(3), N(1)-Ge(1)-C(45) 110.63(14), C(1)-N(1)-Si(1) 119.5(2).

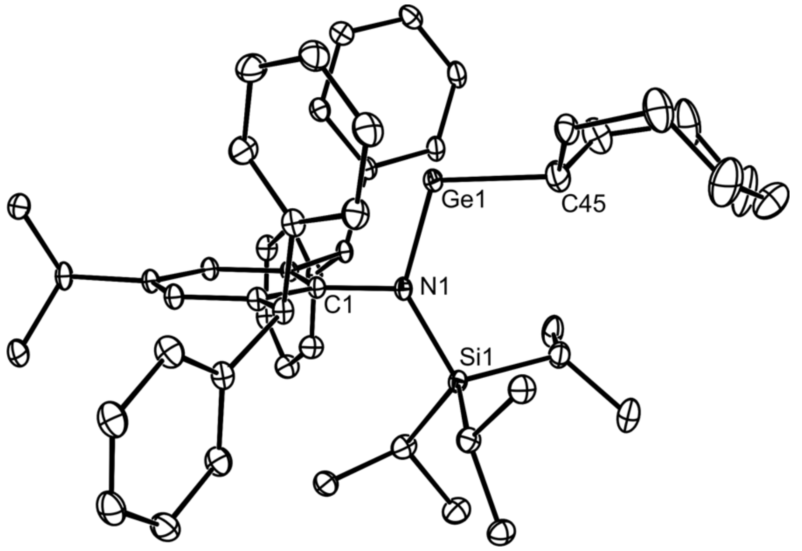

Figure S2. ORTEP diagram of compound **10** (25% thermal ellipsoids; hydrogen atoms omitted). Selected bond lengths (Å) and angles (º): Ge(1)-N(1) 1.898(2), Ge(1)-C(45) 2.054(5), N(1)-Ge(1)-C(45) 109.8(3), C(1)-N(1)-Si(1) 119.72(16).

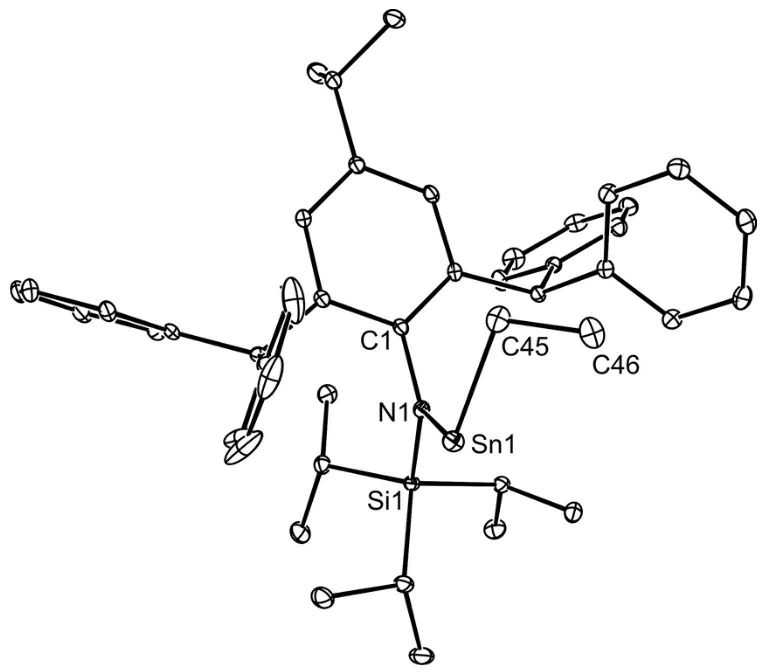

Figure S3. ORTEP diagram of compound **11** (25% thermal ellipsoids; hydrogen atoms omitted). Selected bond lengths (Å) and angles (º): Sn(1)-N(1) 2.123(2), Sn(1)-C(45) 2.182(4), N(1)-Sn(1)-C(45) 101.74(11), Si(1)-N(1)-C(1) 123.80(17).

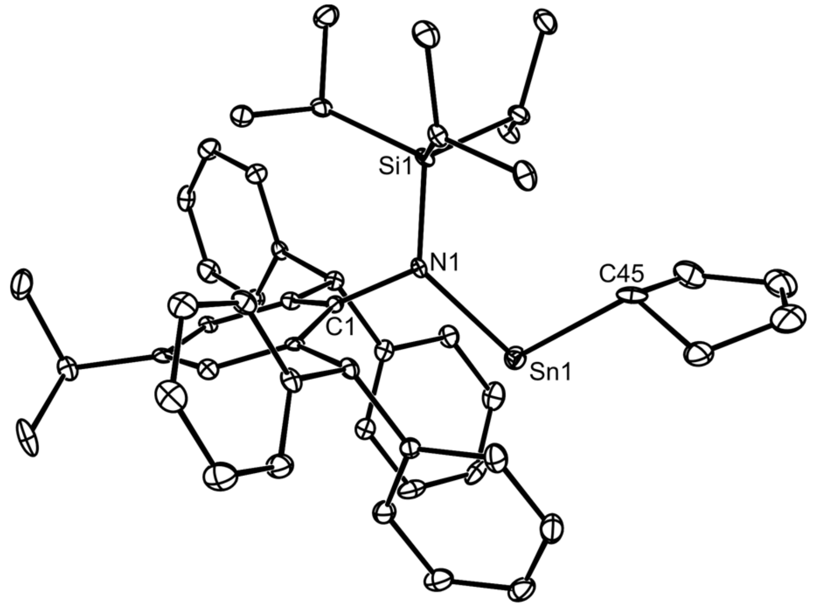

Figure S4. ORTEP diagram of compound **13** (25% thermal ellipsoids; hydrogen atoms omitted). Selected bond lengths (Å) and angles (º): Sn(1)-N(1) 2.127(4), Sn(1)-C(45) 2.168(5), N(1)-Sn(1)-C(45) 102.78(18), C(1)-N(1)-Si(1) 116.2(3).

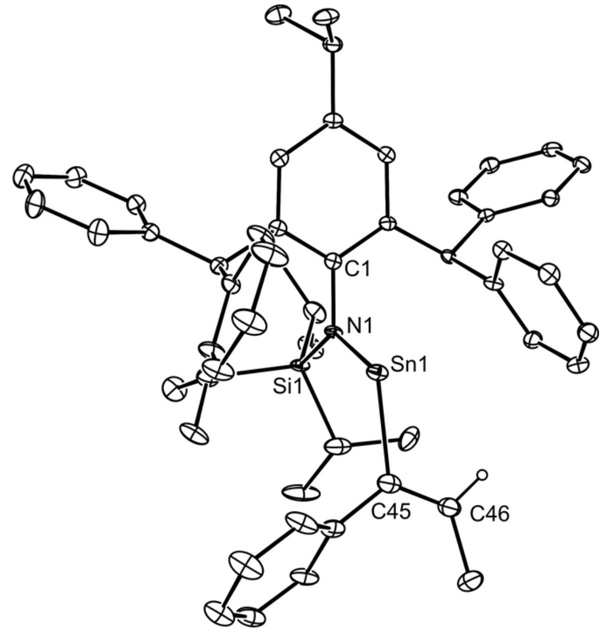

Figure S5. ORTEP diagram of compound **15** (25% thermal ellipsoids; hydrogen atoms omitted). Selected bond lengths (Å) and angles (º): Sn(1)-N(1) 2.122(5), Sn(1)-C(45) 2.229(8), N(1)-Sn(1)-C(45) 107.1(2), C(1)-N(1)-Si(1) 119.5(4).

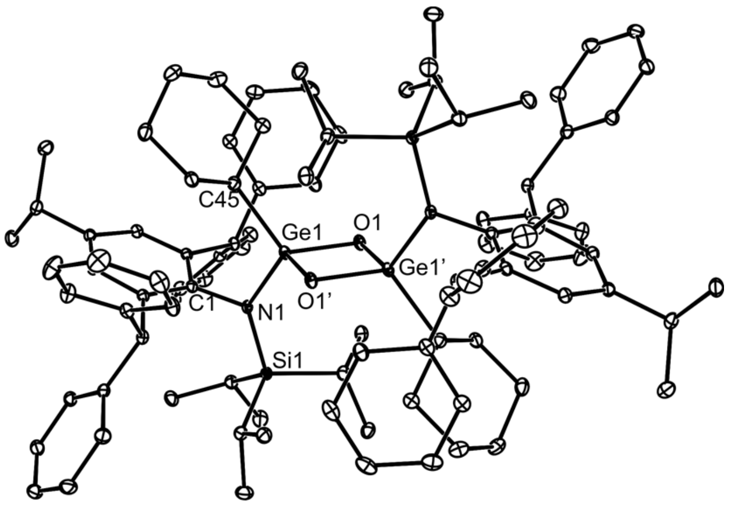

Figure S6. ORTEP diagram of compound **25** (25% thermal ellipsoids; hydrogen atoms omitted). Selected bond lengths (Å) and angles (º): Ge(1)-O(1) 1.8182(14), Ge(1)-O(1)' 1.8288(14), Ge(1)-N(1) 1.8834(17), Ge(1)-C(45) 2.003(2), O(1)-Ge(1)-O(1)' 85.70(6), N(1)-Ge(1)-C(45) 106.24(8), Ge(1)-O(1)-Ge(1)' 94.30(6), C(1)-N(1)-Si(1) 116.46(13).

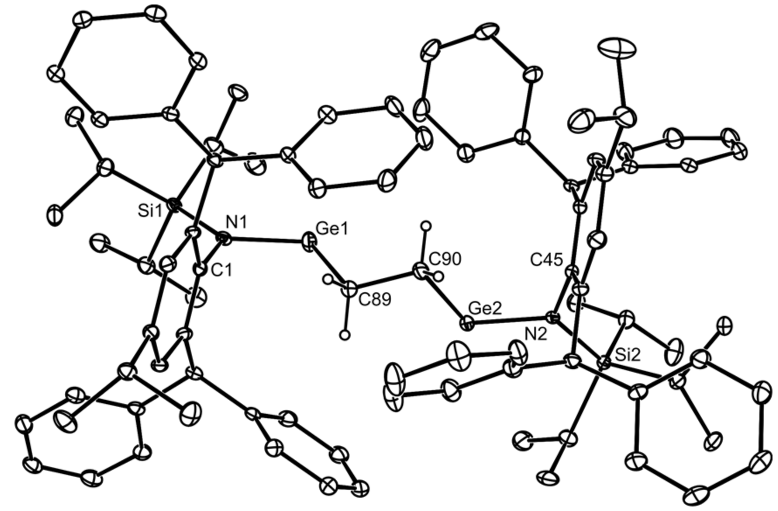

Figure S7. ORTEP diagram of [L†Ge(μ-C_2H_4)GeL†] (25% thermal ellipsoids; hydrogen atoms, except ethylene protons omitted). Selected bond lengths (Å) and angles (º): Ge(1)-N(1) 1.877(3), Ge(1)-C(89) 1.973(4), Ge(2)-N(2) 1.872(3), Ge(2)-C(90) 1.948(5), C(89)-C(90) 1.554(6), N(1)-Ge(1)-C(89) 106.08(16), N(2)-Ge(2)-C(90) 108.55(16), C(1)-N(1)-Si(1) 122.1(2), C(45)-N(2)-Si(2) 124.8(2).

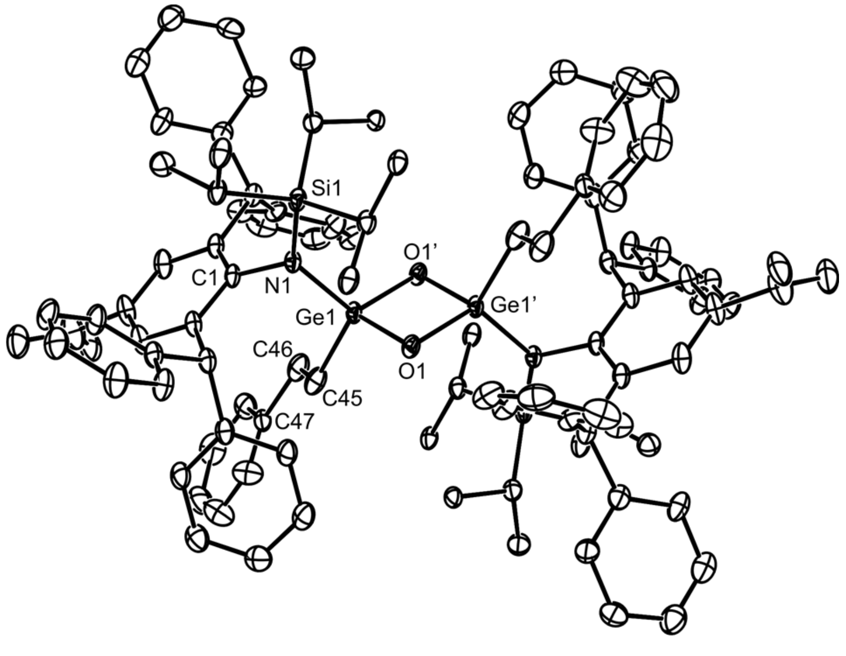

Figure S8. ORTEP diagram of [{L†(PhC_2H_4)Ge(μ-O)}$_2$] (25% thermal ellipsoids; hydrogen atoms omitted). Selected bond lengths (Å) and angles (º): Ge(1)-O(1) 1.802(4), Ge(1)-O(1)' 1.805(3), Ge(1)-N(1) 1.851(4), Ge(1)-C(45) 1.989(8), O(1)-Ge(1)-O(1)' 86.27(17), N(1)-Ge(1)-C(45) 110.5(2), Ge(1)-O(1)-Ge(1)' 93.73(17).

3. Computational Studies

Geometry optimization without symmetry constraints were carried out with the Gaussian 09[8] optimizer in conjunction with Turbomole[9] 6.6 energies and gradients at the TPSS[10] level of theory using def2-TZVPP[11] basis set and applying the dispersion correction by Grimme[12] with Becke-Johnson damping and the RI approximation.[13] Calculations with TPSS on the def2-SVP[14] basis set with the dispersion correction were carried out to characterize stationary points by calculating the Hessian matrix analytically and gain the thermodynamic corrections. Improved single point energies were calculated at the M06-2X[15] level in conjunction with def2-TZVPP basis sets and the dispersion correction by Grimme with the three-body term correction using TPSS/def2-TZVPP optimized geometries.

Table S2. Coordinates and energies of the calculated structures.

Compound 3

100
TPSS+D3(BJ)/def2-TZVPP -4131.064986329

H	-9.2355486	0.0077551	-3.5572628
H	-9.3459647	1.4834885	4.6837969
H	-7.7060540	3.3478921	4.5498222
H	-8.4957446	-1.0339953	-2.3222769
H	-8.3100338	2.3485770	-3.9805181
C	-8.4386498	1.3738947	4.0974966
C	-8.2911437	-0.2660841	-3.0746699
H	-8.3151249	1.3725920	-1.6842216
C	-7.5193567	2.4174541	4.0214983
H	-8.8940400	-0.6356538	3.4577422
H	-7.6377677	-0.7054439	-3.8365407
C	-7.6268123	0.9672769	-2.4367085
C	-8.1840105	0.1848861	3.4095680
C	-7.3736810	2.0597637	-3.4908632
H	-6.9321147	2.9505710	-3.0339426
H	-7.0079241	1.2639254	0.2143162
C	-6.3527300	2.2761652	3.2655183
H	-6.6839106	1.7029575	-4.2630938
H	-6.5296356	-3.3574367	0.5530003
H	-5.6446778	3.0969013	3.2140982
C	-7.0218741	0.0478904	2.6558398
C	-6.3482224	0.5858658	-1.7152042
C	-6.2010324	0.7947271	-0.3426730
H	-5.7704323	-4.2290656	2.6695488
C	-6.0874946	1.0911603	2.5742397
H	-6.8412809	-0.8719570	2.1076921
C	-5.5144063	-3.0867145	0.2336993
H	-5.5883838	-2.2122632	-0.4155038
H	-5.3998255	-4.7013947	-3.7704017
H	-5.3085201	-0.0458810	-3.4852339
H	-5.2136851	-2.4422782	-2.7915962
H	-5.1271935	-3.9174494	-0.3650456
C	-5.2607742	0.0537180	-2.4039471
H	-5.0410025	-1.9535074	1.9933749
C	-4.7189812	-4.0208715	2.4334214
C	-5.0356016	0.4358475	0.3332299

C	-4.6254488	-2.8234490	1.4651267
C	-4.8074503	0.8823217	1.7727043
H	-4.2975860	-4.9299745	1.9936500
C	-4.4428409	-4.1877917	-3.7845182
H	-4.1990379	-3.8356472	3.3767196
H	-4.9784590	3.0930162	0.1637889
C	-4.3407061	-2.9070358	-3.2353118
H	-4.2393141	0.0923696	2.2768279
C	-4.0841778	-0.3463149	-1.7626773
C	-3.8996615	2.1084057	1.7420306
C	-3.9805702	-0.2059418	-0.3601395
C	-4.1376720	3.1596744	0.8463920
H	-3.3095857	-5.2458414	-0.0129523
H	-3.4033635	-5.8047846	-4.7602756
C	-3.3253642	-4.8069499	-4.3393638
H	-2.6267609	1.4254551	3.3431214
C	-2.7938433	2.1994778	2.6006020
H	-3.4979854	-0.9247875	-5.3428105
C	-3.2935819	4.2639359	0.8046654
H	-3.4913866	5.0652331	0.0990523
H	-3.2715929	-2.0806254	4.1680740
C	-3.1198709	-2.2291161	-3.2284441
H	-3.1130743	0.8353276	-7.0350382
C	-3.0294083	0.0097626	-5.0499341
C	-2.9323384	-0.8380192	-2.6306259
Si	-2.8345096	-2.3227611	1.0514917
H	-2.6320096	-3.1884510	-1.1747552
C	-2.8073745	1.0028634	-6.0062113
N	-2.8403950	-0.6575688	0.3833492
C	-2.6466403	0.2028334	-3.7188986
C	-1.9325456	3.3041380	2.5475091
C	-2.1778721	4.3337452	1.6450343
C	-2.2538460	-4.9684693	-0.0796384
C	-2.1928946	2.2007970	-5.6454968
C	-2.0335692	1.4111205	-3.3653962
H	-2.0159832	2.9715449	-6.3897955
H	-2.0130789	-4.3296471	3.3457071
H	-2.7308090	-0.5395716	3.5037397
C	-2.0742820	-3.4506142	-0.2685224
H	-1.8289317	-5.5032731	-0.9381946
C	-1.8046979	2.4001720	-4.3187203
H	-1.7560550	1.5816316	-2.3282712
C	-2.3975763	-1.5403640	3.7901125
H	-1.0740322	3.3467766	3.2105776
H	-1.5090480	5.1873693	1.5953604
C	-2.1013167	-4.1327118	-4.3483368
H	-2.0465691	-0.8883286	-1.9867080
H	-1.7530119	-5.3370885	0.8206973
C	-2.0041568	-2.8568269	-3.8021340
H	-1.3245129	3.3293501	-4.0250540
C	-1.7388864	-2.2747817	2.6099751
H	-1.9465020	1.6293035	-0.2990509
H	-1.6917850	-1.4294445	4.6228057
C	-1.2049912	-3.6465616	3.0666951
Ge	-1.3916959	0.5133563	0.7018942
H	-1.2224979	-4.6043176	-4.7784541
H	-1.0508568	-2.3351839	-3.8102307
H	-0.6115802	-4.1339064	2.2891122
H	-0.8654279	-1.6765243	2.3068963
C	-0.5986449	-3.1016173	-0.5281564
H	-0.5611616	-3.5237429	3.9469285
H	-0.4513714	-2.0240351	-0.6734538
H	-0.2336137	-3.6108431	-1.4281279

H 0.0397474 -3.4061466 0.3080744

M06-2x+D3(ABC)/def2-TZVPP: -4129.81166

Cyclohexene

16
TPSS+D3(BJ)/def2-TZVPP: -234.7907212541
C -1.5530981 0.0667843 -1.3264503
C -1.9620468 1.1887338 -0.7250665
C -2.4457039 1.2395756 0.7014215
C -2.0982714 -0.0444464 1.4684440
C -2.4142261 -1.2824837 0.6200529
C -1.5728144 -1.2869037 -0.6642427
H -1.1901836 0.1120447 -2.3520312
H -1.9449005 2.1272068 -1.2769415
H -2.0103723 2.1122540 1.2052428
H -3.5341723 1.4031985 0.7140391
H -2.6418737 -0.0765343 2.4190115
H -1.0274164 -0.0398867 1.7102835
H -2.2374312 -2.2000369 1.1918160
H -3.4787609 -1.2705202 0.3519888
H -1.9598770 -2.0342177 -1.3689556
H -0.5418803 -1.5996277 -0.4382271

M06-2x+D3(ABC)/def2-TZVPP: -234.61927

TS3-9

116
TPSS+D3(BJ)/def2-TZVPP -4365.848878744
H -1.2620649 3.2191913 -5.5559798
H -0.6415676 1.5987928 -5.1719959
C -1.1665218 2.4678981 -4.7644043
H -2.1738676 2.1479200 -4.4767021
H -1.2173980 5.0603512 -3.7936692
H 3.6981000 -1.1414122 -5.1157227
H 5.8504206 0.1127015 -5.1539142
H 0.5899911 3.3432047 -3.8862367
H -4.1657087 -2.2845467 -3.9307813
C -0.4126712 3.0465838 -3.5532004
C -1.1261648 4.2982198 -3.0121568
H -6.2112121 -0.8665719 -4.0368534
H -2.1348096 4.0493992 -2.6646383
C 4.0099385 -0.5643967 -4.2497359
C 5.2159094 0.1402174 -4.2731043
H 0.6472201 -2.7245832 -3.6057187
C -4.3256917 -1.4996955 -3.1969995
C -5.4718760 -0.7042461 -3.2583168
H -0.5763612 4.7303139 -2.1701805
H 2.2462675 -1.0446387 -3.1282502
C 3.1968925 -0.5233898 -3.1209856
C -0.2340845 1.9996142 -2.4707894
H 1.9046005 1.9700740 -2.5831385
C 0.1324495 -2.6118287 -2.6420184
H -0.7475318 -3.2642238 -2.6744202
H -0.2131470 -1.5773119 -2.5653285
H 2.1074915 -4.4614215 -2.7140408
C 5.5900994 0.8852907 -3.1577429
H -2.3346941 1.7277354 -2.1064554
H -2.4774258 -1.8874714 -2.1821332
H 6.5193901 1.4477457 -3.1644791

C	-3.3788643	-1.2854622	-2.1993017
C	1.0285793	1.5567485	-2.0896065
C	-5.6536192	0.3026429	-2.3126861
H	-6.5363571	0.9344800	-2.3523472
C	-1.3287051	1.4264291	-1.8267448
H	0.8495686	-5.1797780	-1.7045309
C	1.6323027	-4.4158684	-1.7254734
C	3.5702194	0.2100714	-1.9848671
C	4.7740183	0.9191454	-2.0236357
H	1.9255920	-2.3188586	-1.5110613
C	1.0668056	-3.0024724	-1.4817356
C	-3.5553134	-0.2852434	-1.2343098
H	-1.2471167	-4.9944674	-1.2804416
C	-4.7044500	0.5085820	-1.3086180
C	1.2188285	0.5798625	-1.1062352
H	2.3867673	-4.6941370	-0.9852251
C	-1.1897091	0.4585823	-0.8308958
H	5.0787493	1.5070646	-1.1645829
H	1.9089718	2.7321143	0.0941213
H	-4.8611554	1.2957958	-0.5786752
C	2.6545979	0.2047302	-0.7547406
C	0.0997363	0.0048938	-0.4440947
H	-1.7032068	2.5032500	0.5712391
C	-1.5944097	-4.9034165	-0.2478013
H	-2.6479935	-5.2111718	-0.2271875
H	-2.0248317	-2.8343971	-0.4230669
C	-2.4643676	-0.0614775	-0.1812357
H	2.6330917	-0.8252265	-0.3774581
C	2.7195168	2.3217517	0.6871554
H	-1.0322910	-5.6206642	0.3564630
H	-2.2234051	-1.0317757	0.2599678
C	-1.4572318	-3.4613363	0.2782087
Si	0.3032027	-2.7132502	0.2447011
H	3.3094321	-3.4760366	0.5007148
C	3.2138000	1.0496885	0.3857817
C	-2.4901404	2.1018967	1.2007478
N	0.2673736	-0.9794124	0.5902330
H	2.8231069	4.0269112	1.9937247
C	-2.9681332	0.8121641	0.9609154
H	3.0158928	-2.0023457	1.4344305
C	3.2301500	3.0463037	1.7647950
H	1.5226361	-5.5621223	0.8936113
C	2.8865699	-3.0901733	1.4340257
H	-2.6273480	3.8728187	2.4150439
C	-3.0122546	2.8716640	2.2432782
H	-3.1836258	-3.6604781	1.6015414
C	1.4058802	-3.4815333	1.6027002
C	-2.1404187	-3.3225922	1.6494364
C	4.2536243	0.5316510	1.1701854
H	4.6556572	-0.4502142	0.9358418
H	3.4878707	-3.4896182	2.2607475
C	1.2667206	-5.0031074	1.7985252
C	-3.9766583	0.3051941	1.7944202
H	-2.1430965	-2.2845874	1.9963133
H	-4.3582435	-0.6963217	1.6137147
H	0.2498894	-5.2836691	2.0864679
C	4.2450793	2.5098597	2.5556055
Ge	1.0132629	-0.2524480	2.2573591
H	-1.6359567	-3.9279624	2.4113567
H	1.0666305	-3.0172003	2.5390088
C	4.7622442	1.2492487	2.2492010
H	1.9413749	-5.3423942	2.5951373
C	-4.0169530	2.3607230	3.0628325

H	4.6323475	3.0681393	3.4026690
C	-4.4976727	1.0687736	2.8360899
H	-4.4195072	2.9593685	3.8743969
H	5.5564238	0.8234074	2.8554288
H	-0.6249058	-2.0331184	3.4622385
H	-5.2770143	0.6572741	3.4708600
C	-0.1911416	-1.1343245	3.8875590
H	0.4638466	-2.3729216	5.5224109
H	-1.8587844	-0.0279085	3.0791524
C	0.6387093	-1.3530497	5.1613990
H	1.7113743	-1.2857732	4.9433000
C	-0.9739925	0.0550115	3.7017907
H	-0.1900506	0.9078867	2.6033712
H	-0.6458281	-0.6051049	6.7448722
C	0.3103013	-0.3429517	6.2717935
H	-1.9276639	0.9956439	5.3353353
C	-1.0053424	1.1458358	4.7530160
H	1.0783689	-0.3898820	7.0515351
H	-1.0963956	2.1295628	4.2847231
C	0.1989100	1.0729346	5.6958407
H	1.1168298	1.3253636	5.1473439
H	0.0885119	1.8135295	6.4951900

M06-2x+D3(ABC)/def2-TZVPP: -4364.42336

Compound 9

116
TPSS+D3(BJ)/def2-TZVPP -4365.892530425

H	-0.9695229	1.7891030	-6.4728546
H	-0.4423879	0.3381204	-5.5921601
C	-0.9451020	1.3046384	-5.4906733
H	-1.9781541	1.1156298	-5.1792450
H	-0.9281424	4.0689330	-5.3262613
H	5.5461822	-1.7811712	-3.5452547
H	6.6954385	0.3456020	-4.1344433
H	0.8051088	2.3534817	-4.8116810
H	-5.0132571	-1.8898004	-3.6575999
C	-0.2229598	2.1944901	-4.4625215
C	-0.9069897	3.5685339	-4.3521635
H	-6.8473258	-0.2081041	-3.5778700
H	-1.9410008	3.4612639	-4.0068173
C	5.2229841	-0.8444698	-3.1004093
C	5.8691194	0.3478336	-3.4300060
H	0.2321998	-2.6796946	-3.6162963
C	-4.9529878	-1.0484290	-2.9732993
C	-5.9825835	-0.1046333	-2.9293474
H	-0.3791779	4.2140293	-3.6431598
H	3.6695760	-1.7705131	-1.9378280
C	4.1645522	-0.8377458	-2.1934403
C	-0.1366769	1.5040064	-3.1147574
H	2.0090896	1.4467326	-3.0753192
C	-0.2313105	-2.5289789	-2.6329756
H	-1.0695528	-3.2306214	-2.5590802
H	-0.6321269	-1.5141668	-2.6075155
H	1.8483242	-4.2316630	-2.7520543
C	5.4524347	1.5402800	-2.8383951
H	-2.2636305	1.4630621	-2.7790741
H	-3.0382252	-1.6368894	-2.1956822
H	5.9551648	2.4723108	-3.0799782
C	-3.8449033	-0.9115165	-2.1422612

C	1.0966908	1.1744710	-2.5516661
C	-5.8898095	0.9691468	-2.0466102
H	-6.6838107	1.7091003	-2.0038155
C	-1.2872224	1.1891739	-2.3898061
H	0.7292476	-4.9695100	-1.6050622
C	1.4536406	-4.1583700	-1.7303464
C	3.7303509	0.3544613	-1.6019881
C	4.3942557	1.5418850	-1.9290164
H	1.5946926	-2.0247180	-1.6619845
C	0.8058721	-2.7744132	-1.5196968
C	-3.7419977	0.1646430	-1.2492927
H	-1.4181238	-4.8076944	-1.0745575
C	-4.7772575	1.1016640	-1.2112738
C	1.1966526	0.5093101	-1.3278423
H	2.2810021	-4.3456572	-1.0414258
C	-1.2279159	0.5039517	-1.1753328
H	4.0855025	2.4715481	-1.4606397
H	1.5239270	2.8840444	-0.4724161
H	-4.7146579	1.9401398	-0.5249211
C	2.5476605	0.3296578	-0.6441399
C	0.0230136	0.1006392	-0.6542699
H	-1.7079801	2.9161756	-0.3810967
C	-1.8151148	-4.6267578	-0.0720638
H	-2.8844680	-4.8732807	-0.0904723
H	-2.1692306	-2.5501252	-0.3523956
C	-2.5004784	0.2734672	-0.3709954
H	2.5408062	-0.6548696	-0.1684104
C	2.1103184	2.6222959	0.4022727
H	-1.3298820	-5.3309539	0.6088617
H	-2.3860905	-0.6836563	0.1452493
C	-1.6194829	-3.1652590	0.3754765
Si	0.1558334	-2.4715799	0.2569823
H	3.2032365	-3.1886250	0.4062554
C	2.6930267	1.3518662	0.4816204
C	-2.1814788	2.6362201	0.5540867
N	0.0982619	-0.7021580	0.5448290
H	1.7747643	4.5101760	1.3729619
C	-2.6324870	1.3219284	0.7267326
H	2.9128107	-1.7723183	1.4217871
C	2.2433052	3.5334037	1.4482904
H	1.3862766	-5.3294907	0.7894252
C	2.7927003	-2.8565902	1.3651549
H	-1.9348201	4.5798585	1.4363462
C	-2.3026017	3.5684017	1.5814761
H	-3.3352654	-3.1940228	1.7271798
C	1.3188060	-3.2578977	1.5493068
C	-2.2693291	-2.9342521	1.7492398
C	3.4200459	1.0201919	1.6317402
H	3.8952953	0.0460476	1.7002563
H	3.4108933	-3.2990101	2.1569425
C	1.1832841	-4.7844429	1.7153274
C	-3.2233181	0.9691357	1.9476085
H	-2.1826064	-1.8905111	2.0681650
H	-3.5917037	-0.0439029	2.0845353
H	0.1825747	-5.0641056	2.0552279
C	2.9460000	3.1830812	2.6004746
Ge	0.0492233	0.4135838	2.1115800
H	-1.7952893	-3.5518432	2.5204768
H	1.0043551	-2.8217932	2.5018348
C	3.5368390	1.9222854	2.6888507
H	1.8976242	-5.1400309	2.4690444
C	-2.8711251	3.2012482	2.8019630
H	3.0300184	3.8843245	3.4251007

C	-3.3370874	1.8993785	2.9811351
H	-2.9482180	3.9238180	3.6086093
H	4.0865183	1.6380374	3.5812119
H	-0.2860843	-1.9308356	3.4141535
H	-3.7804768	1.6040930	3.9270623
C	-0.0233108	-0.9074305	3.6965833
H	2.1236302	-1.3054248	3.6746945
H	-2.0849658	-0.4792040	4.2396033
C	1.3702103	-0.8960995	4.3576524
H	1.6695806	0.1453442	4.5531142
C	-1.0880603	-0.4012684	4.6878512
H	-0.9258981	0.6685710	4.8915893
H	1.1945984	-2.7356968	5.4757522
C	1.3933554	-1.6746573	5.6823395
H	-1.3415758	-2.2210194	5.8222499
C	-1.0599491	-1.1763450	6.0147653
H	2.3909336	-1.6185792	6.1360908
H	-1.8068677	-0.7671975	6.7068459
C	0.3335266	-1.1417174	6.6536994
H	0.5812332	-0.1047087	6.9213562
H	0.3419598	-1.7204275	7.5849620

M06-2x+D3(ABC)/def2-TZVPP: -4364.47504

4. References

1. T. J. Hadlington, M. Hermann, J. Li, G. Frenking, C. Jones, *Angew. Chem. Int. Ed.*, 2013, **52**, 10389.
2. T. J. Hadlington, C. Jones, *Chem. Commun.*, 2014, **50**, 2321.
3. T. J. Hadlington, M. Hermann, G. Frenking, C. Jones, *J. Am. Chem. Soc.*, 2014, **136**, 3028.
4. N. A. Giffin, M. Makramalla, A. D. Hendsbee, K. N. Robertsson, C. Sherren, C. C. Pye, J. D. Masuda, J. A. C. Clyburne, *Org. Biomol, Chem.*, 2011, **9**, 3672.
5. T.M. McPhillips, S. McPhillips, H.J. Chiu, A.E. Cohen, A.M. Deacon, P.J. Ellis, E. Garman, A. Gonzalez, N.K. Sauter, R.P. Phizackerley, S.M. Soltis, P. Kuhn, *J. Synchrotron Rad.*, 2002, **9**, 401.
6. W.J. Kabsch, *Appl. Cryst.*, 1993, **26**, 795.
7. G.M. Sheldrick, *SHELX-97*, University of Göttingen, 1997.
8. Gaussian 09, Revision C.01, M. J. Frisch, G. W. Trucks, H. B. Schlegel, G. E. Scuseria, M. A. Robb, J. R. Cheeseman, G. Scalmani, V. Barone, B. Mennucci, G. A. Petersson, H. Nakatsuji, M. Caricato, X. Li, H. P. Hratchian, A. F. Izmaylov, J. Bloino, G. Zheng, J. L. Sonnenberg, M. Hada, M. Ehara, K. Toyota, R. Fukuda, J. Hasegawa, M. Ishida, T. Nakajima, Y. Honda, O. Kitao, H. Nakai, T. Vreven, J. A. Montgomery, Jr., J. E. Peralta, F. Ogliaro, M. Bearpark, J. J. Heyd, E. Brothers, K. N. Kudin, V. N. Staroverov, R. Kobayashi, J. Normand, K. Raghavachari, A. Rendell, J. C. Burant, S. S. Iyengar, J. Tomasi, M. Cossi, N. Rega, J. M. Millam, M. Klene, J. E. Knox, J. B. Cross, V. Bakken, C. Adamo, J. Jaramillo, R. Gomperts, R. E. Stratmann, O. Yazyev, A. J. Austin, R. Cammi, C. Pomelli, J.

W. Ochterski, R. L. Martin, K. Morokuma, V. G. Zakrzewski, G. A. Voth, P. Salvador, J. J. Dannenberg, S. Dapprich, A. D. Daniels, ?. Farkas, J. B. Foresman, J. V. Ortiz, J. Cioslowski, and D. J. Fox, Gaussian, Inc., Wallingford CT, 2009.

9. TURBOMOLE V6.6 2014, a development of University of Karlsruhe and Forschungszentrum Karlsruhe GmbH, 1989-2007, TURBOMOLE GmbH, since 2007; available from http://www.turbomole.com.

10. J. Tao, J. P. Perdew, V. N. Staroverov, G. E. Scuseria, *Phys. Rev. Lett.*, 2003, **91**, 146401.

11. (a) F. Weigend, M. Häser, H. Patzelt and R. Ahlrichs; *Chem. Phys. Lett.*, 1998, **294**, 143; (b) F. Weigend, R. Ahlrichs, *Phys. Chem. Chem. Phys.*, 2005, **7**, 3297.

12. (a) S. Grimme, S. Ehrlich, L. Goerigk, *J. Comput. Chem.*, 2011, **32**, 1456; (b) S. Grimme, J. Antony, S. Ehrlich and H. Krieg, *J. Chem. Phys.*, 2010, **132**, 154104.

13. (a) K. Eichkorn, O. Treutler, H. Ohm, M. Häser, R. Ahlrichs, *Chem. Phys. Lett.*, 1995, **242**, 652; (b) F. Weigend, R. Ahlrichs, *Phys. Chem. Chem. Phys*., 2006, **8**, 1057; (c) F. Weigend, M. Häser, *Theor. Chem. Acc*., 1997, **97**, 331; (d) F. Weigend, M. Häser, H. Patzelt, R. Ahlrichs, *Chem. Phys. Lett*., 1998, **294**, 143.

14. A. Schäfer, H. Horn, R. Ahlrichs, *J. Chem. Phys.*, 1992, **97**, 2571.

15. Y. Zhao and D. G. Truhlar, *Theor. Chem. Acc.*, 2006, **120**, 215.
